# Mid-Infrared Photons Alleviate Tinnitus by Activating the KCNQ2 Channel in the Auditory Cortex

**DOI:** 10.34133/research.0479

**Published:** 2024-09-18

**Authors:** Peng Liu, Xinmiao Xue, Chi Zhang, Hanwen Zhou, Zhiwei Ding, Li Wang, Yuke Jiang, Zhixin Zhang, Weidong Shen, Shiming Yang, Fangyuan Wang

**Affiliations:** ^1^ Senior Department of Otolaryngology Head and Neck Surgery, the 6th Medical Center of Chinese PLA General Hospital, Chinese PLA Medical School, Beijing 100853, China.; ^2^ State Key Laboratory of Hearing and Balance Science, Beijing 100853, China.; ^3^ National Clinical Research Center for Otolaryngologic Diseases, Beijing 100853, China.; ^4^ Key Laboratory of Hearing Science, Ministry of Education, Beijing 100853, China.; ^5^ Beijing Key Laboratory of Hearing Impairment Prevention and Treatment, Beijing 100853, China.

## Abstract

Tinnitus is a phantom auditory sensation often accompanied by hearing loss, cognitive impairments, and psychological disturbances in various populations. Dysfunction of KCNQ2 and KCNQ3 channels—voltage-dependent potassium ion channels—in the cochlear nucleus can cause tinnitus. Despite the recognized significance of KCNQ2 and KCNQ3 channels in the auditory cortex, their precise relationship and implications in the pathogenesis of tinnitus remain areas of scientific inquiry. This study aimed to elucidate the pathological roles of KCNQ2 and KCNQ3 channels within the auditory cortex in tinnitus development and examine the therapeutic potential of mid-infrared photons for tinnitus treatment. We utilized a noise-induced tinnitus model combined with immunofluorescence, electrophysiological recording, and molecular dynamic simulation to investigate the morphological and physiological alterations after inducing tinnitus. Moreover, in vivo irradiation was administered to verify the treatment effects of infrared photons. Tinnitus was verified by deficits of the gap ratio with similar prepulse inhibition ratio and auditory brainstem response threshold. We observed an important enhancement in neuronal excitability in the auditory cortex using patch-clamp recordings, which correlated with KCNQ2 and KCNQ3 channel dysfunction. After irradiation with infrared photons, excitatory neuron firing was inhibited owing to increased KCNQ2 current resulting from structural alterations in the filter region. Meanwhile, deficits of the acoustic startle response in tinnitus animals were alleviated by infrared photons. Furthermore, infrared photons reversed the abnormal hyperexcitability of excitatory neurons in the tinnitus group. This study provided a novel method for modulating neuron excitability in the auditory cortex using KCNQ2 channels through a nonthermal effect. Infrared photons effectively mitigated tinnitus-related behaviors by suppressing abnormal neural excitability, potentially laying the groundwork for innovative therapeutic approaches for tinnitus treatment.

## Introduction

Tinnitus is a phantom sensation characterized by intermittent or continuous perception of sound in the ear or head, without external acoustic stimuli [1]. Tinnitus affects approximately 10% to 15% of the global population, with approximately one-tenth experiencing severe symptoms, including cognitive impairments, sleep disturbances, anxiety, depression, and concentration difficulties [2,3]. Despite their prevalence, effective treatments for these auditory hallucinations remain elusive, necessitating improved therapeutic approaches. Tinnitus often coincides with hearing loss, primarily attributed to changes in neural plasticity along the auditory pathway from the periphery to the brain [4]. However, attempts to alleviate tinnitus through cochlear nucleus injury or auditory nerve transection have proven ineffective, suggesting the indispensable role of higher brain regions, such as the auditory cortex, in tinnitus development and maintenance [5,6].

Previous studies have shown a marked increase in regional homogeneity of the auditory cortex and a significant decrease in cortical volume and surface area after tinnitus development, highlighting the important role of the auditory cortex in tinnitus [7]. Moreover, α-training stimulation can significantly increase the regional homogeneity of the right frontal lobe and relieve tinnitus symptoms [8]. Furthermore, genes related to neurodegenerative diseases changes correlate with tinnitus [9].

The auditory cortex exhibits increased excitability after tinnitus onset, characterized by elevated spontaneous discharge in individual neurons, synchronous activity among neighboring neurons, and a reconfigured tonotopic map [10–14]. However, the precise mechanism underlying this increased cortical neuronal excitability after tinnitus onset remains elusive. Clarifying the mechanism of altered neuronal excitability in the auditory cortex is crucial for identifying targets for tinnitus treatment.

KCNQ2 and KCNQ3 channels are important voltage-dependent potassium ion channel subtypes that regulate neuronal excitability [15,16]. These ion channels exhibit slow activation and inactivation during action potential (AP) firing, effectively reducing AP frequency [17]. Dysfunction of KCNQ2 and KCNQ3 channels results in increased neuronal excitability, contributing to various neurological diseases [18]. Moreover, dysfunction of KCNQ2 and KCNQ3 channels in the cochlear nucleus can cause tinnitus [19]. However, the precise correlation between KCNQ2 and KCNQ3 channels within the auditory cortex and the etiology of tinnitus have not yet been fully elucidated. Numerous studies have attempted to utilize KCNQ2/3 channel activators, such as retigabine, or systemically administered newly synthesized molecules to alleviate tinnitus [20]. Despite their significant efficiency, certain adverse effects, such as urinary retention and retinal abnormalities, necessitate a novel approach to tinnitus treatment [21].

With technological advancements, diverse physical neuromodulations using magnetic, electrical, and optical methods have been applied to regulate neural excitability and treat neurological disorders [22]. Optical neuromodulation, particularly near-infrared stimulation, has garnered increasing attention owing to its high spatial precision in the brain. The thermal effect induced by near-infrared radiation results in regulatory effects through heat generation, which is a consequence of the vibrational activity of intracellular polar molecules and the direct absorption of near-infrared energy by the physiological liquid environment. This increase in temperature subsequently activates heat-sensitive ion channels, altering cellular membrane permeability [23].

Mid-infrared photons, a subtype of electromagnetic wave frequencies of 12 to 120 THz, affect biological processes [24]. The specific frequencies of infrared photons can modulate neuronal excitability by influencing the structure of ion channels on cell membranes, resulting in excitatory or inhibitory effects [22,25,26]. Infrared photon therapy has been tested for various neurological and psychiatric conditions, with the potential to improve cognitive impairment associated with posttraumatic stress disorder by regulating hippocampal neurotransmission [27]. Additionally, infrared photons influence cancer cell migration and survival by altering chromatin accessibility and telomerase activity [28,29].

In this study, we explored the pathological roles of KCNQ2 and KCNQ3 channels in the auditory cortex in tinnitus development, emphasizing the crucial role of hyperexcitability of excitatory neurons. We hypothesized that mid-infrared photons can alleviate tinnitus by rectifying abnormal excitability while preventing side effects of systemic drug delivery. Moreover, we aimed to verify the therapeutic potential of infrared photons in tinnitus treatment by precisely implanting fibers in the auditory cortex. Our study offers insights for developing effective tinnitus treatments, focusing on accurately modulating excitatory interneurons in the auditory cortex with high security, adjustability, and temporal-spatial resolution.

## Results

### Establishment and verification of noise-induced tinnitus mouse model

**Fig. 1.**
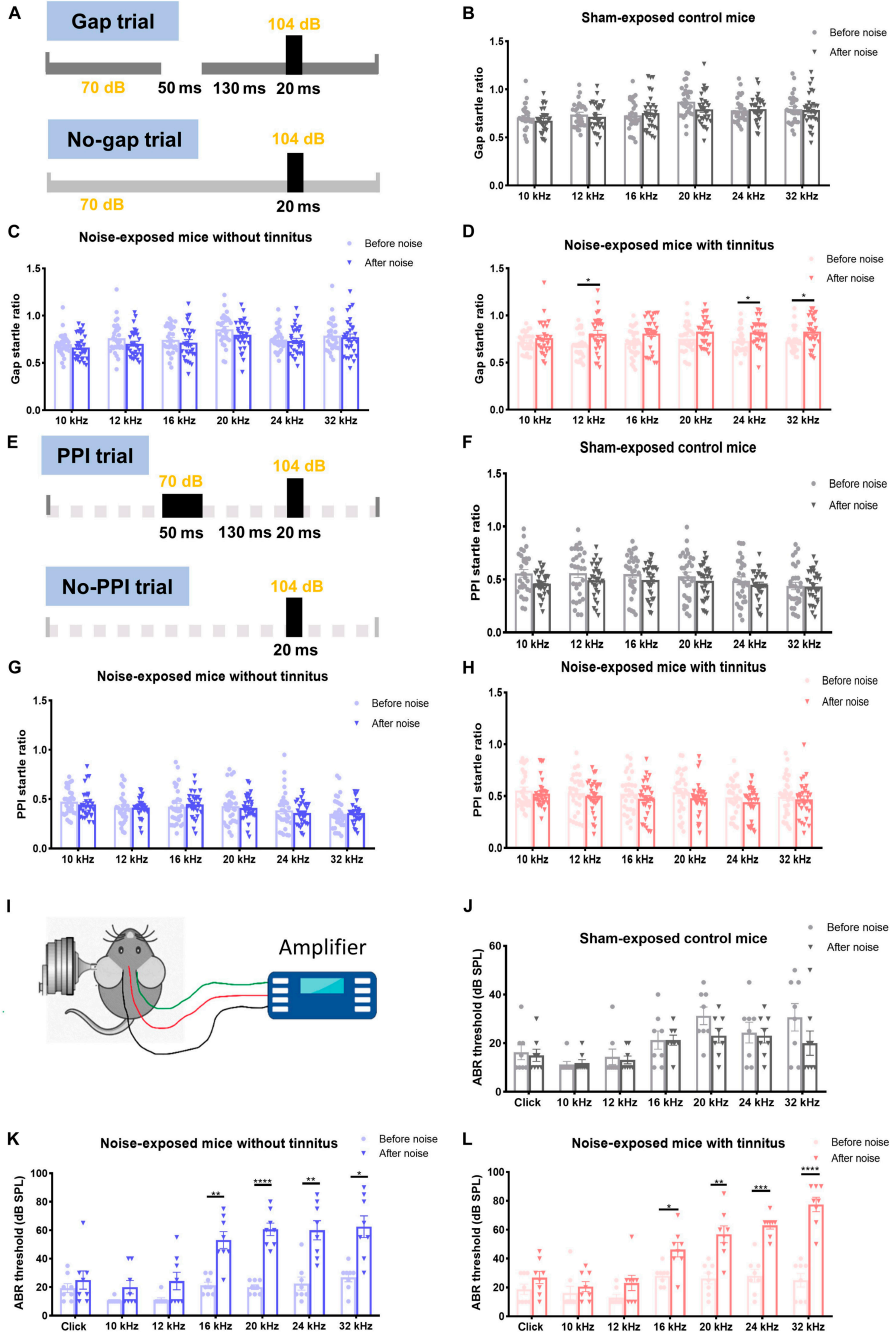
Verification of noise-induced tinnitus. (A) Experimental paradigm for gap startle ratios. (B to D) Gap startle ratios in the control (B), nontinnitus (C), and tinnitus groups (D). (E) Experimental paradigm for PPI startle ratio. (F to H) PPI startle ratios in the control (F), nontinnitus (G), and tinnitus (H) groups. (I) Experimental paradigm for auditory brainstem response. (G to L) ABR thresholds in the control (G), nontinnitus (K), and tinnitus (L) groups. *N* = 30 mice; *P < 0.05; error bars indicate SEM.

Previous studies have induced tinnitus in animals using loud noise exposure [19,30]. Mice were exposed to 116-dB sound pressure level (SPL) narrowband noise, and their acoustic startle responses were assessed using gap detection tasks to establish a noise-induced tinnitus model (Fig. 1A). Mice that met the criteria for tinnitus were assigned to the tinnitus group, whereas those that did not meet the criteria were assigned to the nontinnitus group. The gap startle ratios before and after noise exposure did not significantly differ between the control and nontinnitus groups across all frequencies (Fig. 1B and C). However, a significant increase in the gap startle ratio was observed at 12, 24, and 32 kHz in the tinnitus group (Fig. 1D), indicating that tinnitus-related behavior occurred at these frequencies.

The prepulse inhibition (PPI) startle ratio was calculated for the control, nontinnitus, and tinnitus groups to exclude potential temporal processing deficits or behavioral problems [31] (Fig. 1E). No significant differences were observed in the ratios before and after noise exposure between the control and nontinnitus groups across all frequencies (Fig. 1F and G). Similarly, the ratio in the tinnitus group remained consistent across all frequency bands following noise exposure (Fig. 1H), suggesting that tinnitus-related behaviors were not attributable to temporal processing deficits.

The auditory brainstem response (ABR) threshold in the left ear was measured across all groups to assess the impact of hearing loss on gap detection (Fig. 1I). No significant changes were observed in the ABR threshold for click or tone frequencies in the control group (Fig. 1J). No significant differences were observed in the left ear thresholds for click, 10 kHz, and 12 kHz before and after noise exposure in the nontinnitus group. However, a significant elevation in the hearing threshold was observed at 16, 20, 24, and 32 kHz (Fig. 1K). Similarly, hearing ability remained unaffected at clicks, 10 kHz, and 12 kHz in the tinnitus group but exhibited impairment at higher frequencies, including 16, 20, 24, and 32 kHz (Fig. 1L). These findings showed a parallel change in the ABR threshold between the tinnitus and nontinnitus groups, suggesting that deficits in GAP detection were attributable to tinnitus rather than hearing loss.

### Noise-induced tinnitus activated excitatory glutamatergic neurons instead of parvalbumin-positive inhibitory neurons

**Fig. 2.**
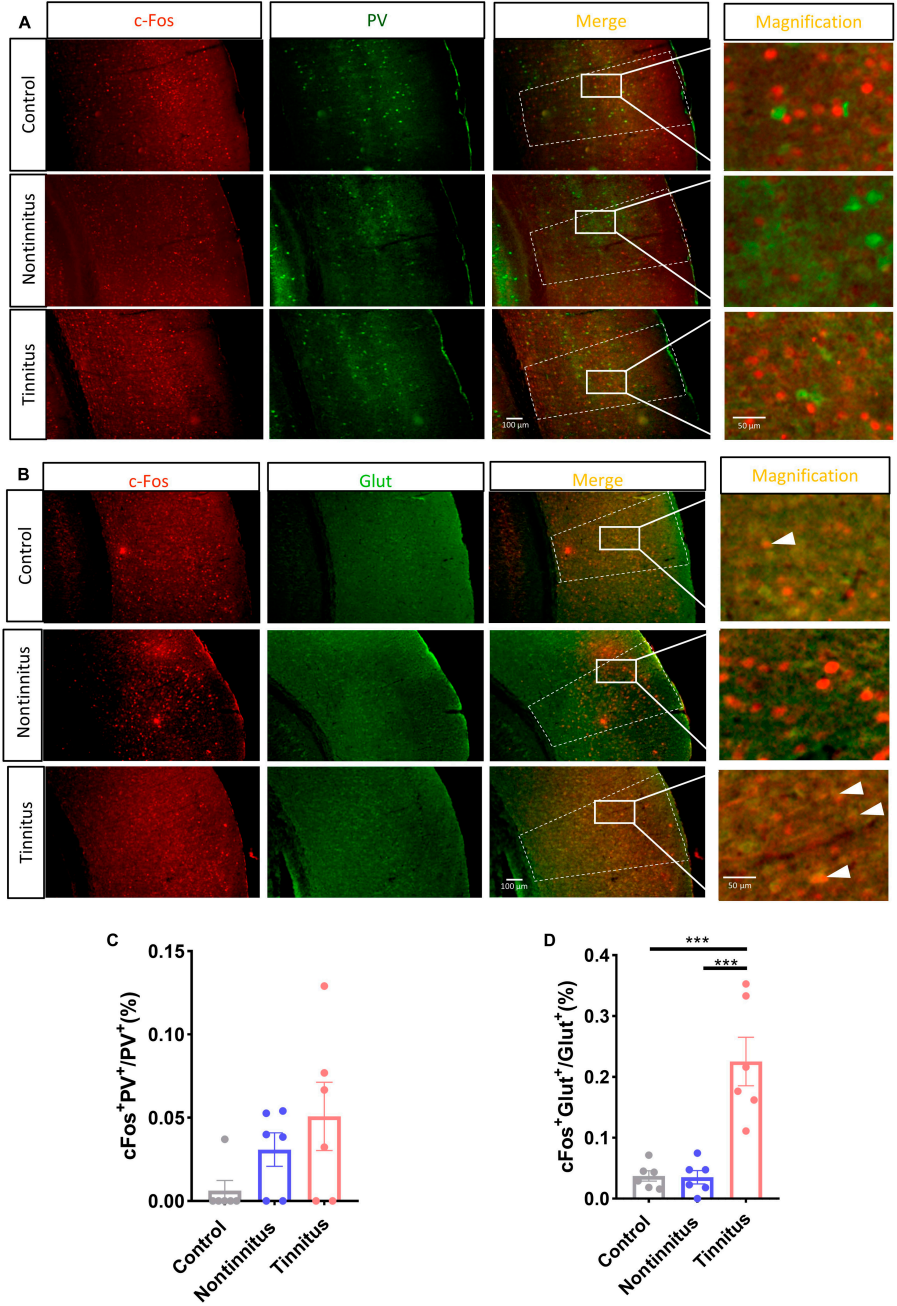
Tinnitus increases the activation of excitatory neurons instead of PV-positive neurons. (A) Activated PV-positive neurons in the control (upper), nontinnitus (middle), and tinnitus (lower) groups. (B) Activated excitatory neurons in the control (upper), nontinnitus (middle), and tinnitus (lower) groups. (C) Activation ratio of PV-positive neurons. (D) Activation ratio of excitatory neurons. *N* = 6 slices; ***P < 0.001; error bars indicate the SEM.

We examined neural activation in the auditory cortex to determine the involvement of specific neuron subtypes in tinnitus-related behaviors. c-Fos staining revealed that parvalbumin (PV)-positive neurons showed no significant activation in the tinnitus group compared to the nontinnitus and control groups (Fig. 2A and C). However, the activation ratio of excitatory interneurons was significantly higher in the tinnitus group than in the nontinnitus and control groups (Fig. 2B and D). Golgi staining and electron microscopy showed that primary neurons in the auditory cortex exhibited increased dendritic complexity and excitatory synapses and larger postsynaptic densities and active zones after tinnitus induction (Figs. S1 and S2). These findings indicated heightened activation of excitatory pyramidal neurons in the auditory cortex following noise-induced tinnitus.

### Tinnitus altered synaptic transmission between excitatory neurons in the auditory cortex

**Fig. 3.**
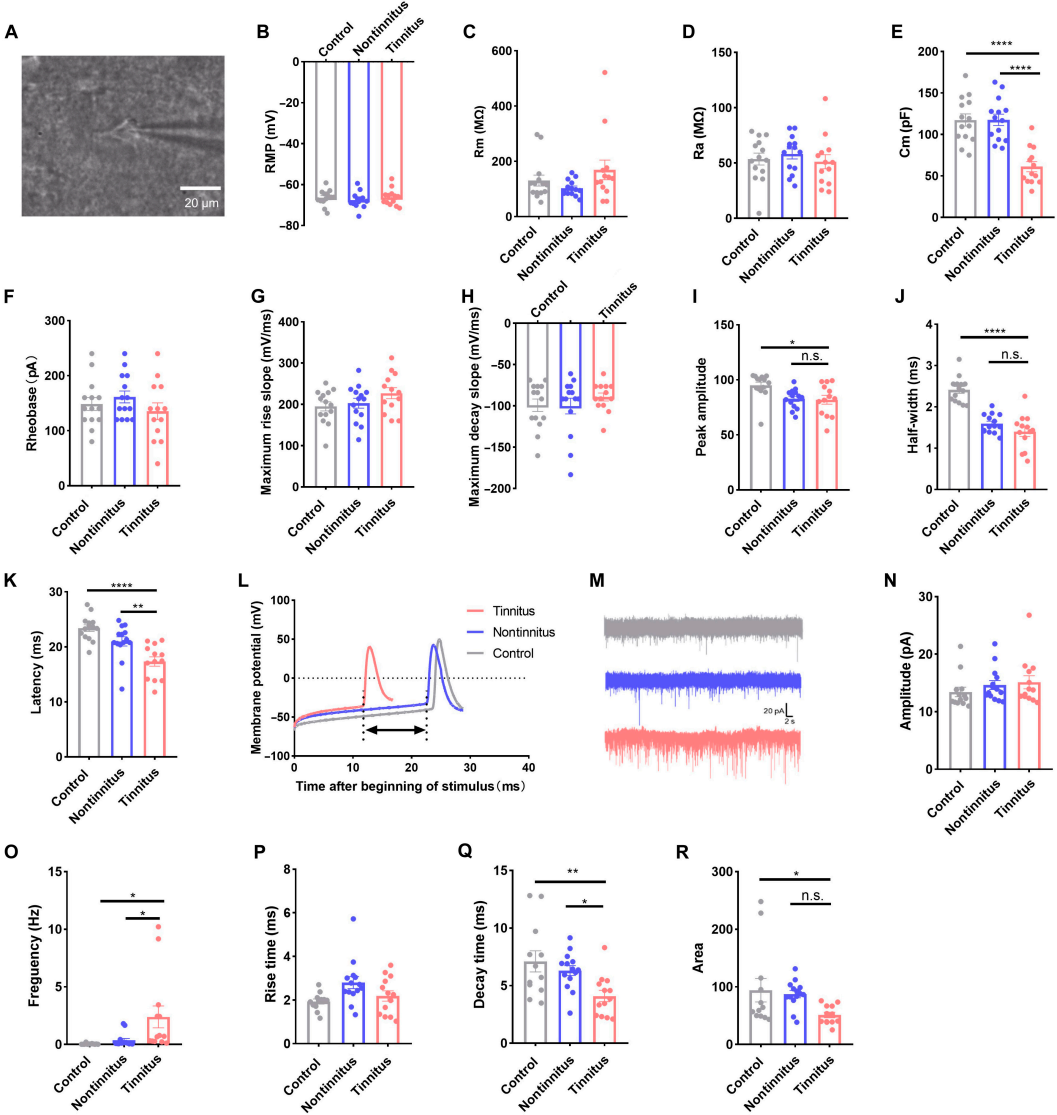
Influence of tinnitus on passive and active membrane properties and sEPSCs of pyramidal neurons in the auditory cortex. Example of a patched cell in the auditory cortex (A). (B to G) Comparisons of passive membrane properties, including RMP (B), Rm (C), Ra (D), Cm (E), and rheobase (F), and active membrane properties, including maximum rise (G), decay (H) slope, peak amplitude (I), half-width (J), and latency (K and L) among control, nontinnitus, and tinnitus groups. (M) Representative data of sEPSCs in the control, nontinnitus, and tinnitus groups. (N to R) Summary of amplitude, frequency, rise and decay times, and area for 3 groups are shown in (N), (O), (P), (Q), and (R), respectively. *N* = 13 to 14 cells; *P < 0.05, **P < 0.01, ***P < 0.001; error bars indicate SEM.

In vitro patch-clamp recordings of neurons in the auditory cortex were conducted to identify whether functional changes occurred in excitatory neurons after tinnitus induction (Fig. 3A). The resting membrane potential (RMP) of pyramidal neurons did not exhibit a statistically significant change in the tinnitus group (Fig. 3B), nor did access resistance (Ra) and membrane resistance (Rm) (Fig. 3C and D). However, membrane capacitance (Cm) showed a significant decrease in the tinnitus group compared to the control and nontinnitus groups (Fig. 3E). Membrane potential responses to negative current injections were provided, and input resistance was not statistically significant among the groups (Fig. S3). No significant differences in active membrane properties were observed between the tinnitus and nontinnitus groups, including AP rheobase (Fig. 3F), maximum rise slope (Fig. 3G), maximum decay slope (Fig. 3H), amplitude (Fig. 3I), and half-width (Fig. 3J). However, AP latency (Fig. 3K and L) significantly decreased in the tinnitus group compared to the control and nontinnitus groups. Moreover, spontaneous excitatory postsynaptic currents (sEPSCs) were examined since synaptic properties reflect neuronal function (Fig. 3M). The sEPSC amplitudes were not significantly different among the control, nontinnitus, and tinnitus groups (Fig. 3N). However, sEPSC frequency significantly increased in the tinnitus group, indicating heightened excitatory input to pyramidal neurons from presynaptic neurons (Fig. 3O). The kinetics of each sEPSC was also analyzed. The rise time was not statistically significant (Fig. 3P); however, the decay time was shorter in the tinnitus group (Fig. 3Q). The sEPSC area in the tinnitus group was comparable to that in the nontinnitus group (Fig. 3R).

Considering the strong correlation between AP latency and KCNQ2 and KCNQ3 channels [32], the observed shorter latency in the tinnitus group may be attributed to abnormal functioning or expression of KCNQ2 and KCNQ3 channels. However, RNA sequencing, immunoblotting, and immunostaining analyses revealed comparable transcription and translation levels among the tinnitus, control, and nontinnitus groups, suggesting that the hyperexcitability associated with tinnitus is likely mediated by abnormal KCNQ2 and KCNQ3 functioning rather than aberrant protein expression (Fig. S4).

### Infrared photons inhibited the excitability of excitatory neurons in the auditory cortex through KCNQ2 channels

**Fig. 4.**
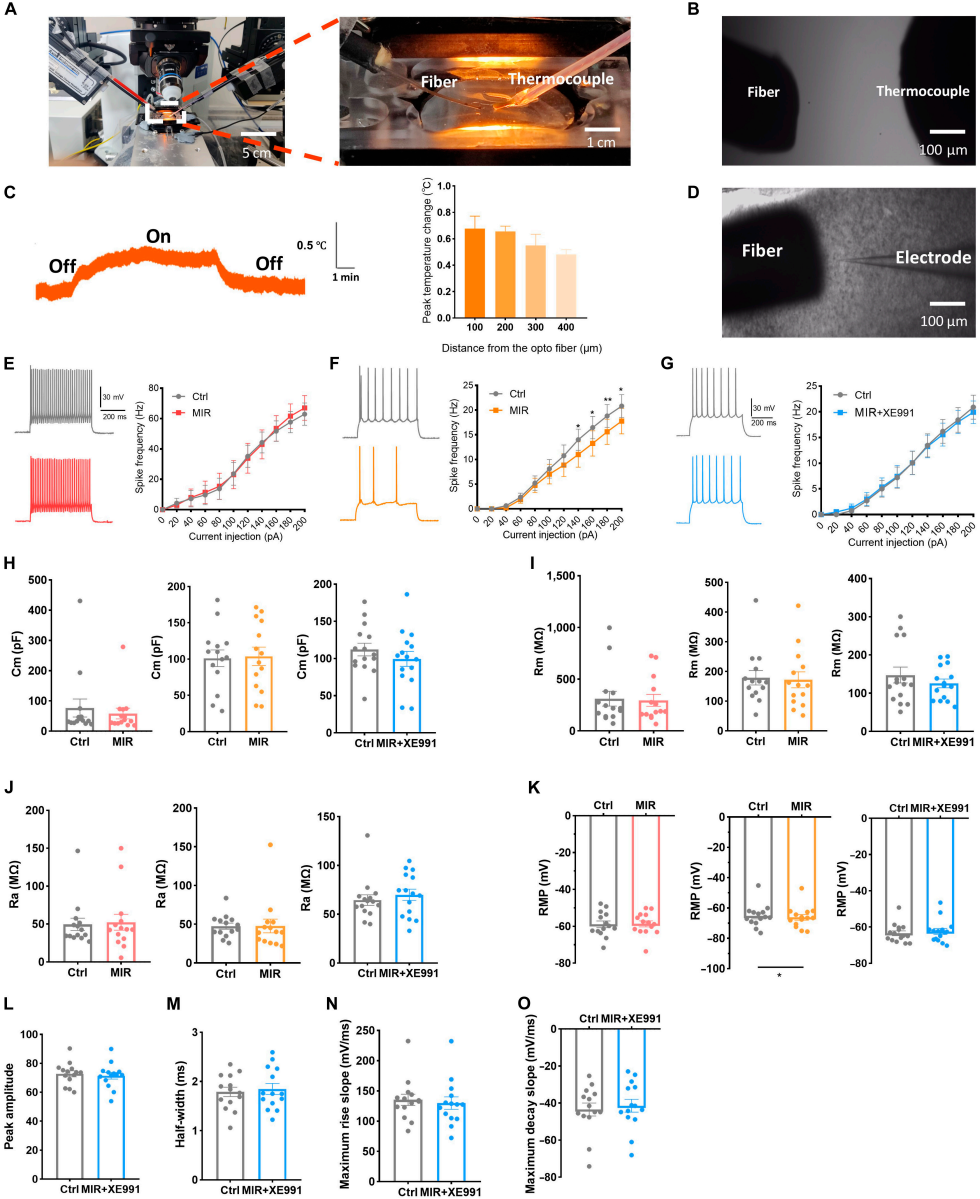
Inhibitory effects of mid-infrared (MIR) on the excitatory neurons in the auditory cortex. (A and B) Picture of the MIR irradiation apparatus. (C) Thermal change with respect to the lateral distance from the fiber tip. (D) The relative position between fiber and electrode. (E) AP firing of inhibitory neurons between control and MIR. (F) Similar to (E) but belonging to excitatory neurons. (G) AP firing of excitatory neurons between MIR and MIR+XE991. (H to K) Passive membrane properties of neurons among the control, MIR, and MIR+XE991. (L to O) Active membrane properties of excitatory neurons between MIR and MIR+XE991. *N* = 14 to 15 cells, *P < 0.05, **P < 0.01, ***P < 0.001; error bars indicate SEM.

We conducted patch-clamp recordings in the auditory cortex during infrared photon irradiation to determine whether infrared photons modulate neuronal excitability. We first measured temperature changes near the neurons during infrared photon illumination to exclude thermal effects on neural excitability (Fig. 4A and B). The temperature change decreased with increasing distance between the fiber and temperature probe (Fig. 4C). These findings indicated no significant temperature increase during infrared photon irradiation [27]. Next, we investigated whether infrared photons could modulate the firing of different neurons (Fig. 4D). Infrared photons did not affect inhibitory neuron firing but significantly suppressed excitatory neuron firing (Fig. 4E and F). This effect was abolished by administering XE991, an antagonist of KCNQ2 and KCNQ3 (Fig. 4G).

Additionally, infrared photons did not affect the passive membrane properties of inhibitory neurons but increased the RMP of excitatory neurons, which can be inhibited by the XE991 (Fig. 4H to K). Meanwhile, they had no effect on active membrane properties (Fig. 4L to O). Furthermore, infrared photon irradiation did not affect neuron morphology or KCNQ2 and KCNQ3 channel expression (Figs. S5 to S7). These findings suggested that infrared photons can inhibit neuron excitability through KCNQ2 and KCNQ3 in the auditory cortex.

### Infrared photons promoted K^+^ efflux from neurons by loosening the filter region of the KCNQ2 channel

**Fig. 5.**
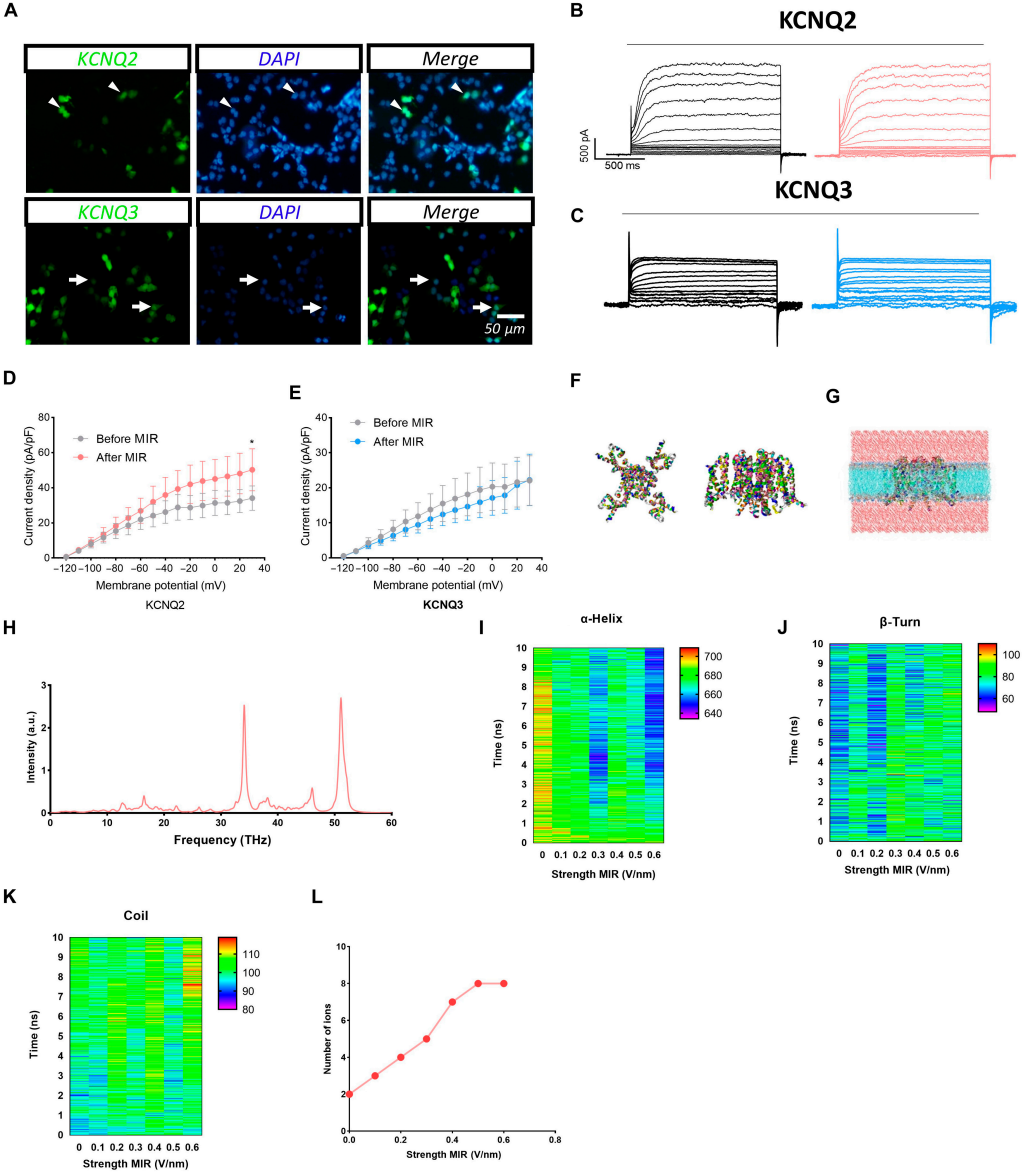
Regulatory effect of MIR on KCNQ2 and KCNQ3 channels.(A) Image of transfected HEK293 cell with KCNQ2 and KCNQ3 plasmid. (B and C) Representative current traces of KCNQ2 and KCNQ3 channels. (D and E) Representative current density–voltage curve of KCNQ2 and KCNQ3 channels. (F and G) The concrete structure of the KCNQ2 protein. (H) Optimal absorption frequency of the KCNQ2 channel. (I to K) The changes in protein secondary structure of KCNQ2 with different field strengths. (L) The number of ions passing through the KCNQ2 channel. *N* = 5 cells, *P < 0.05, **P < 0.01, ***P < 0.001; error bars indicate SEM. DAPI, 4′,6-diamidino-2-phenylindole.

We tested the current densities of the KCNQ2 and KCNQ3 channels to identify the channel mediating the inhibitory effect of infrared photons on neural excitability. Infrared photons significantly reduced the current density of KCNQ2 but not that of KCNQ3, as shown in Fig. 5A to E. These findings suggested that infrared photon irradiation inhibits neuronal excitability by modulating KCNQ2 channel function.

To further elucidate the specific mechanism underlying the increase in current density, we conducted molecular dynamics simulations of the KCNQ2 channel using the Protein Data Bank (7CR0) structure to simulate its state after infrared photon exposure (Fig. 5F and G). The simulations revealed that the optimal frequency for the KCNQ2 channel was 34.09 THz, our experimental condition (Fig. 5H). Additionally, we examined changes in the protein’s secondary structure and observed a decrease in α-helix content, coupled with an increase in β-turn and coil structures, with increased electromagnetic field intensity (Fig. 5I to K). Moreover, the number of ions passing through the KCNQ2 channel increased following exposure to the electromagnetic photon field, stabilizing when the field intensity exceeded 0.5 V/nm (Fig. 5L). These findings suggested that infrared photons inhibit excitatory neuron excitability by altering the filter region and promoting K^+^ efflux through KCNQ2.

### Infrared photons alleviated tinnitus by inhibiting excitatory neurons in the auditory cortex by promoting KCNQ2 channel opening

**Fig. 6.**
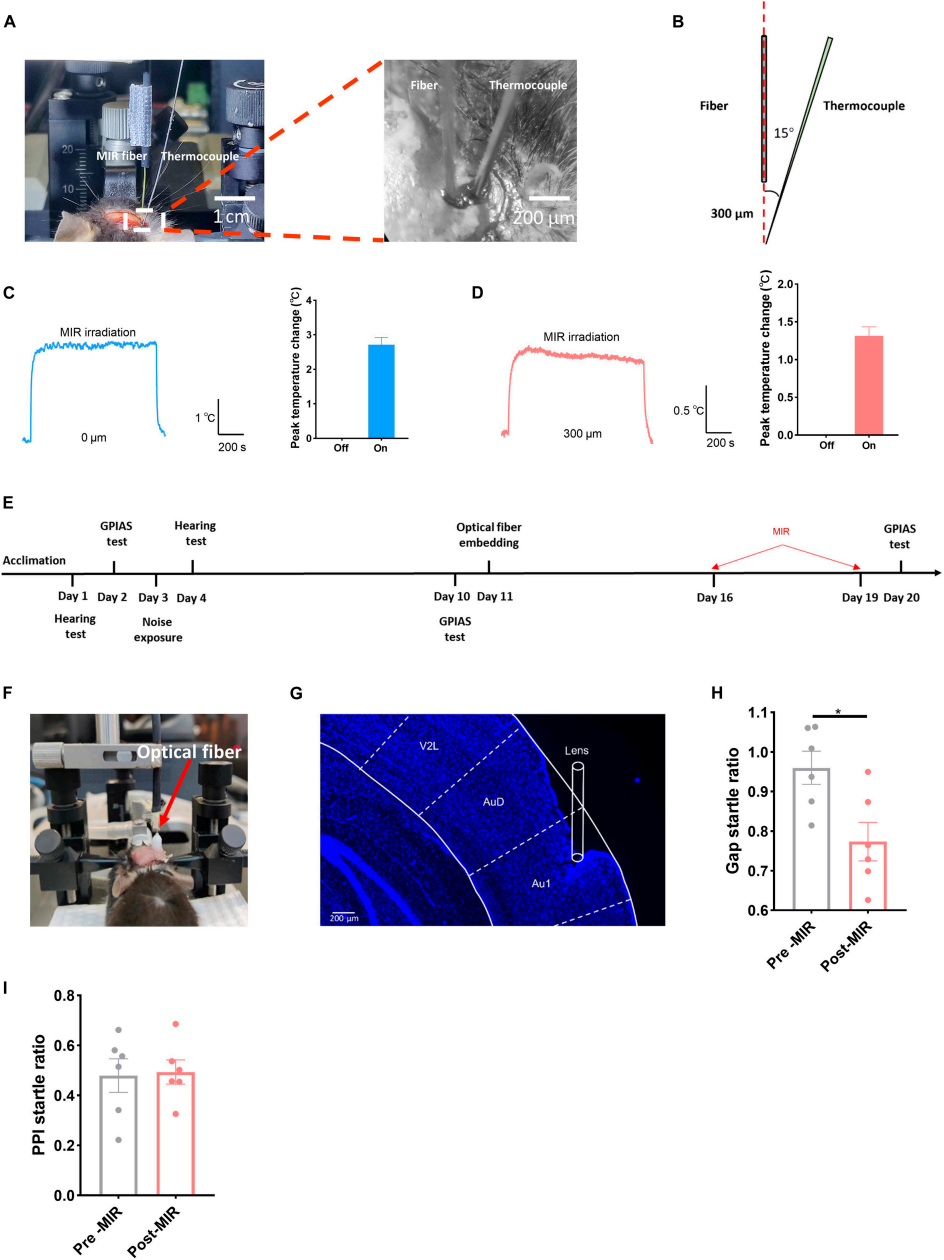
Inhibitory effects of MIR on the tinnitus. (A and B) MIR irradiation apparatus. (C) Thermal change of tissue at the fiber tip. (D) Thermal change of tissue at the position of 300 μm below the fiber tip. (E) Timeline of experimental design. (F) Image showing MIR irradiation in vivo. (G) The relative depth of fiber in the auditory cortex. (H) The gap startle ratio changes before and after MIR irradiation. (I) PPI startle ratio change before and after MIR irradiation. *N* = 6 mice, *P < 0.05, **P < 0.01, ***P < 0.001; error bars indicate SEM.GPIAS, gap prepulse inhibition of acoustic startle response.

Next, we examined whether the inhibitory effects of infrared photons can alleviate tinnitus. Similar to the abovementioned experiment, we initially assessed the temperature change after applying infrared photons in vivo. The maximum temperature change of the target area was less than 2 °C, which has no significant impact on brain neurons according to previous research (Fig. 6A to D) [33–36]. The gap startle ratio of tinnitus mice was significantly inhibited by infrared photon exposure, with no effect on the PPI startle ratio (Fig. 6E to I).

**Fig. 7.**
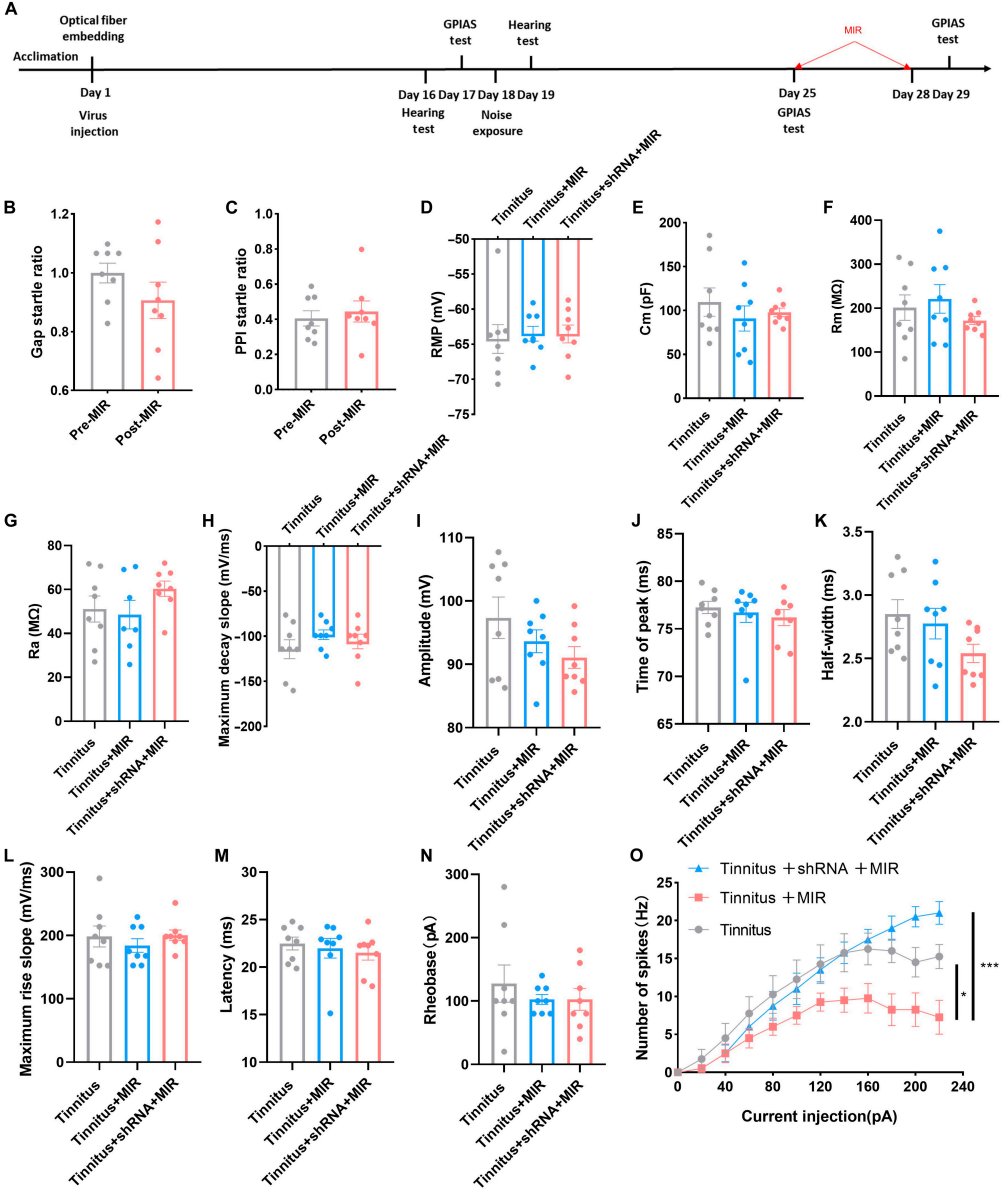
KCNQ2 virus inhibits the treatment effect of MIR on tinnitus. (A) Timeline of experimental design. (B) Gap startle ratio change before and after MIR irradiation in the tinnitus mice with KCNQ2 virus. (C) PPI startle ratio change before and after MIR irradiation in the tinnitus mice with KCNQ2 virus. (D to G) Passive membrane properties of excitatory neurons in tinnitus, tinnitus with MIR, and tinnitus with MIR injected with virus groups. (H to O) Active properties of excitatory neurons in tinnitus, tinnitus with MIR, and tinnitus with MIR injected with virus groups. *N* = 8 mice for behavioral test, and *N* = 8 cells for patch-clamp recording. Error bars indicate SEM.

We applied recombinant adeno-associated viral (rAAV) vectors encoding a KCNQ2-specific short hairpin RNA (rAAV-shKCNQ2) virus capable of inhibiting KCNQ2 channel expression to verify that the therapeutic effect of infrared photons occurs through the KCNQ2 channel. The virus’s expression and functionality were validated before the main experiments (Figs. S8 and S9). Inhibiting KCNQ2 expression abolished the therapeutic effect of infrared photons on tinnitus (Fig. 7A to C). Next, we compared neuronal excitability among the tinnitus group without infrared photons, the tinnitus group with infrared photons, and the tinnitus group with infrared photons injected with the virus. No significant changes were observed in the neurons’ passive and active membrane properties among the 3 groups (Fig. 7D to N); however, we observed that infrared photons could inhibit neuron spiking in tinnitus mice. Still, KCNQ2 knockdown abolished this effect (Fig. 7O). These findings suggested that THz can alleviate tinnitus by inhibiting excitatory neurons through KCNQ2 channels.

## Discussion

Various methods, including transcranial magnetic and deep brain stimulation, have been explored for tinnitus modulation; however, these modalities lack satisfactory efficiency owing to their unclear mechanisms [37]. Despite extensive literature highlighting auditory cortex hyperactivity in tinnitus, researchers have yet to identify effective interventions to modulate this abnormal neuronal excitability. Recent advancements in neuromodulation using optogenetics have demonstrated precise control over neuronal firing; however, this technology raises concerns regarding potential risks associated with introducing exogenous genetic material [38]. Our findings may lay the groundwork for developing novel medications targeting excitatory neurons in the auditory cortex, offering relief from the physiological and psychological burden experienced by patients with refractory tinnitus.

In this study, we initially investigated functional alterations in excitatory neurons within the auditory cortex of noise-induced tinnitus mice. Pyramidal neurons in the auditory cortex exhibited important activation in the tinnitus group, consistent with previous findings [39]. The arborization and synaptic connections of neurons are crucial for their ability to receive information from adjacent or distant brain regions. An increase in dendritic branches enhances neuronal capacity to more efficiently process and integrate complex signals [40]. KCNQ2 and KCNQ3 channels, subtypes of voltage-dependent potassium channels, exhibit slow activation and lack inactivation during the entire AP firing process, enabling them to effectively regulate neuronal excitability [41]. In this study, we observed decreased latency of APs in pyramidal neurons in tinnitus animals, indicating KCNQ2 and KCNQ3 channel dysfunction. Additionally, increased sEPSC frequency confirms a higher probability of neural connections owing to increased firing of presynaptic cells [42].

Drug-induced tinnitus typically lasts <3 d and is not directly comparable to chronic tinnitus, which lasts for a significantly longer duration. Moreover, a significant proportion of patients diagnosed with tinnitus report a history of loud noise exposure, whether from recreational, industrial, or military sources. In this study, we used a noise-induced tinnitus model to investigate neural plasticity in the auditory cortex, which has potential implications for clinical practice. Our findings revealed that approximately 50% of C57BL/6J mice exhibited tinnitus-related behaviors, consistent with Li et al.’s findings [43]. This finding aligns with the fact that not all individuals exposed to similar noisy environments develop tinnitus. Tinnitus behaviors were predominantly observed in high-frequency regions (24 and 32 kHz), consistent with previous findings [44]. However, we also detected tinnitus behaviors in low-frequency regions (12 kHz), which did not entirely correspond to the frequency regions associated with hearing loss. This discrepancy may arise because audiograms may not accurately reflect subtle peripheral damage, resulting in a weak correlation between audiogram results and tinnitus pitch [45,46]. Tinnitus behaviors, as reflected by the gap deficit, were not influenced by hearing loss, despite hearing loss commonly preceding the gap deficit [47].

In our study, the activation ratio of PV-positive neurons in the tinnitus group was similar to that in the nontinnitus group, suggesting that PV neurons may play a role in the pathophysiology of hearing loss rather than tinnitus in the auditory cortex, as previously reported [48]. Another explanation for this inconsistency may be the different experimental paradigms applied in our study, resulting in a much shorter exposure time, albeit at the same frequency. Spine density was comparable; however, the number of mushroom spines was higher in the tinnitus group than in the nontinnitus group, indicating more abundant signal exchange among neurons since this subtype processes information more efficiently [49].

Depending on photon power and wavelength, infrared photons can either enhance synaptic transmission by increasing firing in delayed neurons or disrupt neural connections by altering their morphology and firing [50–52]. In this study, infrared photons inhibited excitatory neuron spiking by promoting K^+^ flux, consistent with previous findings [22,53]. However, the inhibitory interneurons (including PV^+^ cells) also express voltage-gated K channels [54]. Infrared photons did not exert similar effects on inhibitory interneurons, which can be attributed to different AP latencies between inhibitory and pyramidal neurons. Inhibitory interneurons have a shorter AP latency than pyramidal neurons [55]; therefore, their KCNQ2 and KCNQ3 channels may be less liable to be influenced to limit dramatic K^+^ flux. Voltage-gated K channels can be expressed in both excitatory and inhibitory neurons; however, inhibitory neurons may have a lower expression. Infrared photons may modulate KCNQ2 channels in inhibitory neurons, but this effect may not necessarily reach an important effect and change the firing. This inhibition was not attributed to thermal effects since temperature did not importantly increase [56]. Previous studies showed that 34.5 THz can modulate potassium functions by altering the structure of the filtering region; however, these studies did not distinguish between specific channel subtypes [53]. In this study, we focused on the KCNQ2 channel, which is strongly associated with tinnitus. Moreover, 34.09 THz, which is close to the utilized frequency, enhances ion flux and regulates excitability, broadening the application of THz radiation with various frequencies. Alleviation of tinnitus by infrared photons was confirmed by the decreased gap startle ratio in the tinnitus group, which could be blocked by KCNQ2 channel inhibition. We confirmed that KCNQ2 in the auditory cortex can alleviate tinnitus development and maintenance. Existing literature on KCNQ2 focused on tinnitus development, rather than treatment [57,58]. In contrast, our study provided insights into tinnitus alleviation, which is crucial for future research in the field.

Major complications of invasive deep brain mid-infrared stimulation include bleeding and inflammation, which may interfere with neuronal function [59]. The PPI ratio remained unchanged, indicating the safety of mid-infrared radiation, as it is an indicator of the impact of complications on time processing [44]. Previous research indicated that tinnitus may be partially alleviated by applying transcranial electrical and magnetic stimulation. However, conflicting findings have been reported, with some studies failing to demonstrate significant efficacy in tinnitus reduction, potentially owing to the variability in stimulation parameters [60–62]. Our study offers a concrete paradigm for the utilization of mid-infrared photons in tinnitus treatment, thereby expanding the therapeutic applications of infrared technology. The accurate modulation of KCNQ2 in the target area prevents systemic drug diffusion, which can cause side effects in other systems. Particularly, the nonthermal effect can enhance the utilization of mid-infrared photons in tinnitus treatment, thereby preventing excessive heating of the target tissue.

This study had some limitations. First, the auditory region receives projections from remote regions, such as the limbic system, which is highly correlated with emotional disorders including anxiety and depression, which are often associated with tinnitus [63,64]. Further studies are required to clarify the extra-auditory roles of pyramidal neuronal activation in the auditory cortex after tinnitus onset. Moreover, considering that KCNQ2 channel function is associated with cell membrane molecules, further studies are required to verify whether the effect of infrared photons is also derived from indirect molecular effects such as phosphatidylinositol 4,5-bisphosphate [65]. Chronic tinnitus is the most uncomfortable tinnitus subtype for patients; therefore, investigating whether THz treatment can alleviate symptoms in chronic subtypes is crucial. Moreover, the efficacy of mid-infrared stimulation should be validated in humans depending on current deep brain stimulation systems, replacing the electrode with a mid-infrared optical fiber. The target area of modulation can be precisely controlled by the image system. Consequently, the translatability aspect of this work may be achieved in the future with advancements in portable laser devices. Finally, whether the efficacy of mid-infrared photon therapy in alleviating tinnitus is sustained remains to be validated.

In conclusion, we demonstrated a significant increase in pyramidal neuron excitability in the auditory cortex using a noise-induced tinnitus model, as evidenced by the heightened frequency of APs and sEPSCs. This phenomenon can be attributed to KCNQ2 and KCNQ3 channel dysfunction. Furthermore, tinnitus-related behaviors exhibited alleviation upon exposure to infrared photons, which facilitate the generation of a K^+^ current due to the loosening of the KCNQ2 channel’s structure. These findings suggested that targeting pyramidal neurons in the auditory cortex with infrared photons may be a promising therapeutic strategy to alleviate refractory tinnitus.

## Materials and Methods

### Animals

Male C57BL/6J mice aged 2 months were maintained under specific-pathogen-free conditions with a 12-h light–dark cycle and provided with ad libitum access to food and water. All procedures were conducted in accordance with the guidelines for the Care and Use of Laboratory Animals of the Chinese PLA General Hospital (Beijing, China) and were approved by the Animal Care Committee of the Chinese PLA General Hospital.

### Auditory brainstem response

ABR was assessed to evaluate hearing threshold shifts in the animals [66,67]. Briefly, the mice were anesthetized with 1% to 1.5% isoflurane and placed in a soundproof chamber. Acoustic stimuli with a 0.5-ms rise/fall were directly delivered to the external auditory canal using a TDT RZ6 instrument (Tucker Davis Technologies). Needle electrodes (Rochester Elektro-Medical, Lutz, FL, USA), comprising reference, grounding, and active electrodes, were subcutaneously positioned over the head before testing [9]. After averaging 512 times and filtering from 100 to 3 kHz, the amplified responses from the active electrodes were recorded. The intensity of the stimuli was decreased from 90 dB in 10-dB SPL increments for both click and different tone frequencies (10, 12, 16, 20, 24, and 32 kHz). The ABR threshold criterion was the lowest intensity at which the repeatable wave II at the respective frequency appeared with decreased SPL.

### Detection of gap and PPI

The startle ratio from the gap detection tasks was computed to verify successful tinnitus induction in noise-exposed mice [19]. Animals were positioned on a platform in a soundproof cage with a piezoelectric transducer beneath the floor. This setup allowed the detection of pressure changes on the floor caused by the startle sound, and voltage transformation was used to determine the amplitude of the acoustic startle response. In the “No-gap” detection paradigm, a 70-dB SPL narrowband noise (centered at 10, 12, 16, 20, 24, and 32 kHz) was paired with a 104-dB SPL startle stimulus (20 ms). The only distinction between the “Gap” and “No-gap” detection paradigms was the presence of a sound gap (lasting 50 ms), delivered 130 ms before the onset of the startling sound in the former. Paired “Gap” and “No-gap” trials at the same frequency were randomly arranged. The gap startle ratio, calculated as the ratio of the average startle amplitude of the “Gap” and “No-gap” trials, assessed the animals’ ability to detect the sound gap. The PPI paradigm was the inverse of the gap detection task, with the sound gap replaced by a nonstartle sound (50 ms, 70-dB SPL) occurring 130 ms before the startling sound (20 ms, 104-dB SPL). Similar to the PPI paradigm, no prepulse (nonstartle sound) was induced in the startle-only trial session. The PPI startle ratio, calculated as the ratio of the average startle amplitude of the prepulse trials to that of the startle-only trials, evaluated the animals’ responses. Mice exhibiting an increased gap startle ratio (>0.3) following noise exposure at any of the frequencies (10, 12, 16, 20, 24, and 32 kHz) were classified as tinnitus animals; otherwise, they were categorized as nontinnitus animals [30,44,68].

### Noise exposure

Normal hearing is essential for detecting sound gaps; therefore, unilateral noise exposure was administered to prevent complete hearing loss [30]. The animals were anesthetized with 1% to 1.5% isoflurane and fitted with foam earplugs (OHRFRIEDEN, Wehrheim, Germany) in the right ear before being placed in a soundproof chamber equipped with a sound delivery system. A TW67 speaker (Pyramid Car Audio, Brooklyn, NY, USA), connected to an RA 300 amplifier (Alesis, Cumberland, RI, USA) was positioned 10 cm from the head. Continuous noise, centered at 16 kHz, was delivered for 1 h at 116-dB SPL.

### Preparation of brain slices

Mice were anesthetized with 1.5% isoflurane and perfused with ice-cooled, preoxygenated artificial cerebrospinal fluid composed of 230 mM sucrose, 2.5 mM KCl, 1.22 mM Na_2_HPO_4_, 24 mM NaHCO_3_, 10 mM MgSO_4_, 0.5 mM CaCl_2_, 10 mM glucose, and 2 mM sodium pyruvate, adjusted to pH 7.3 to 7.4 with a mixture of 95% O_2_ and 5% CO_2_. Subsequently, the whole brain was carefully excised, and the auditory cortex tissue was isolated following the guidelines of the Allen Brain Atlas. Coronal slices (300 μm thick) for electrophysiological recordings were obtained using a vibratome (VT1000S, Leica Microsystems, Deerfield, IL, USA). The tissue slices were subsequently incubated at 33 °C for 1 h in a recovery solution containing 126 mM NaCl, 2.5 mM KCl, 1.25 mM Na_2_HPO_4_, 26 mM NaHCO_3_, 2 mM MgCl_2_, 2 mM CaCl_2_, 10 mM glucose, and 2 mM sodium pyruvate, adjusted to pH 7.3 to 7.4.

### Electrophysiological recordings of brain slices

The brain slices were transferred to an incubation chamber and immersed in a recording solution containing 115 mM NaCl, 5 mM KCl, 1.25 mM Na_2_HPO_4_, 10 mM glucose, 2 mM pyruvate, 25 mM NaHCO_3_, 2 mM MgCl_2_, and 2 mM CaCl_2_, adjusted to pH 7.3 to 7.4 at room temperature (RT) and continuously oxygenated with 95% O_2_ and 5% CO_2_. The target area was identified using infrared differential interference contrast optics (10× magnification). Subsequently, the objective lens was switched to a 40× magnification to locate active cells in the auditory cortex. Glass electrodes (5 to 7 MΩ) were filled with a solution containing 130 mM potassium gluconate, 10 mM KCl, 10 mM Hepes, 1 mM MgCl_2_, 5 mM EGTA, 1 mM CaCl_2_, 2 mM Na_2_ATP, and 0.5 mM Na_3_GTP, adjusted to pH 7.4 (all chemicals from Fluka, NY, USA), and were used for signal detection. Whole-cell recordings were conducted using Multiclamp 700 B amplifiers (Molecular Devices, USA) and a 1550s A/D board (Molecular Devices, USA).

Before normal recording, neuronal membranes with a standard tight seal were punctured under negative pressure. Passive properties, such as RMP, Cm, Rm, Ra, and rheobase, were recorded and analyzed. Concurrently, active properties were determined, including AP peak amplitude, half-width, peak time, maximum rise, and decay slope [69]. Active AP properties were assessed from the first induced AP by incrementally increasing the step current by 20 pA from 0 to 300 pA over 500 ms. The threshold value was defined as the point of significant deflection in positive voltage. The amplitude was quantified as the variance between the peak voltage and threshold. The half-width was delineated as the temporal duration spanning the half-rising and half-decaying slopes of the AP. The maximal rates of depolarization and repolarization were determined by calculating the ratio of the amplitude differentials during rise and decay to the corresponding durations of depolarization and repolarization. The rheobase, which is the minimal current necessary to evoke AP initiation, was ascertained. Additionally, by maintaining the potential at −70 mV, sEPSCs lasting for 5 min were recorded. Postsynaptic current features, including frequency, area, amplitude, and kinetics (rise and decay time constants), were measured using the Mini Analysis software program (Synaptosoft, Decatur, GA, USA), with the event threshold set at 10 pA.

### Preparation of cultured cells

Plasmids encoding human Kv7.2 (AF110020) and rat Kv7.3 (AF091247.1) in a GV658 vector were used for transfection and expression in human embryonic kidney 293 (HEK293) cells. Before transfection, the HEK293 cells were cultured in Dulbecco’s modified Eagle medium (Gibco: 12430104) supplemented with 10% fetal bovine serum (Gibco: 10099141C) and 1% penicillin and streptomycin (Gibco: 15140122). Subsequently, 100 μl of OPTI-MEM (Gibco: 11058021) was mixed with 10 μl of plasmid and 3 μl of Lipofectamine 3000 reagent (Gibco: L3000001), and the mixture was allowed to stand for 20 min. The cultured cells were removed from the incubation box and washed twice with phosphate-buffered saline buffer, and 2 ml of antibiotic-free medium was added. This mixture was then carefully and slowly added to the medium, and the cells were incubated for 5 h. Subsequently, the medium was replaced with Dulbecco’s modified Eagle medium containing fetal bovine serum supplemented with penicillin and streptomycin.

### Electrophysiological recordings of cultured cells

A recording solution (adjusted to pH 7.4) containing 160 mM NaCl, 2.5 mM KCl, 1 mM MgCl_2_, 2 mM CaCl_2_, and 10 mM glucose was used for the cultured cell recordings. Following incubation in the recording chamber, transfected cells were verified using fluorescence microscopy. Subsequently, an electrode containing an internal solution (150 mM KCl, 5 mM MgCl_2_, and 10 mM Hepes, adjusted to pH 7.4) was used to seal the cells with fluorescence. A step voltage ranging from −120 to 30 mV, with an increasing step of 10 mV, was subsequently applied to detect KCNQ2 and KCNQ3 currents.

### Immunofluorescence staining

The mice were anesthetized with 1.5% isoflurane, perfused with cold saline, and fixed in 4% paraformaldehyde for tissue staining. The brain, including the auditory cortex, was isolated and postfixed in the same fixative for 12 h, followed by immersion in 30% sucrose for 48 h. Tissue slices (30 mm) were obtained using a cryotome and preblocked with 5% donkey serum and 0.3% Triton X-100 for 2 h at RT. The sections were subsequently incubated with the following primary antibodies for 12 h at 4 °C: rabbit anti-glutamate (1:500, Sigma-Aldrich, G6642), mouse anti-PV (1:500, Swant, #235), and guinea pig anti-c-FOS antibody (1:500, SYSY, 226308). Subsequently, the tissue slices were incubated with the secondary antibodies, donkey anti-rabbit 488 (1,500, Abcam, Cambridge, ab150073) and donkey anti-guinea pig 594 (1,500, Yeasen, 34512ES60) antibodies, for 2 h at RT. Finally, the slices were photographed and viewed using a confocal microscope (Leica, DMIL).

### Molecular dynamics

Molecular dynamic simulations were conducted using the Gromacs 2018 software package [70]. The dynamics model was built using charm-gui to incorporate the structure of the channel proteins into the dipalmitoyl phosphatidylcholine membrane, supplemented with the transferable intermolecular potential 3 water model and 0.15 mmol of KCl [71]. A spherical cutoff of 1.2 nm was used for van der Waals and short-range Coulomb interactions using the particle-mesh Ewald method. The system’s temperature and pressure were regulated using a Nosé–Hoover thermal thermostat and Berendsen barostat [72]. First, the system’s energy was gradually minimized to regulate the initial molecule velocity. Subsequently, the Nosé–Hoover thermostat and Parrinello–Rahman barostat (NPT) ensemble, using the leapfrog integrator with a time step of 1.0 fs, were utilized for a 1-ns simulation at 300 K for pre-equilibration. Finally, a 10-ns simulation at 300 K was performed using the Nosé–Hoover thermostat (NVT) ensemble with a time step of 2.0 fs to investigate the changes in potassium ion channels across the membrane and alterations in secondary protein structures under various infrared photon intensities. Changes in the secondary structure during dynamic simulations were analyzed using the DSSP software package, and snapshot plots were visualized using VMD software [73].

### Stereotaxic surgery and virus injection

The animals were anesthetized with isoflurane and positioned on the stereotaxic frame (5%). Following the skin removal, the scalp was exposed. Craniotomies were performed above the target brain region using a dental drill. The coordinates for the target area (primary auditory cortex) were determined based on previous literature (anteroposterior axis: −4.2 mm, mediolateral axis: ± 2.65 mm, dorsoventral axis: −2.65 mm) [74]. A 10-μl microsyringe (Hamilton 80384) was used to inject rAAV-CaMKIIa-EGFP-5’miR-30a-shRNA (kcnq2)-3’miR-30a-WPREs (200 nl) into the right auditory cortex at a rate of 50 nl/min. The microsyringe remained in the injected site for an additional 10 min before being slowly withdrawn to prevent overflow. Regarding fiber ferrule implantation, the fiber ferrule (RWD Life Science Co., Ltd., 62003) was aligned with the bregma after leveling, and craniotomies were performed. The fiber ferrule was then slowly positioned in the auditory cortex and secured using dental cement.

### Mid-infrared source

A quantum cascade mid-infrared laser with a constant wavelength of 8.8 μm was utilized for in vivo irradiation. The mid-infrared fiber (core diameter: 240 ± 15 μm, numerical aperture: 0.3 ± 0.03) was coupled with stable power (7.5 ± 0.5 mW). The configuration of mid-infrared utilized includes the pulse duration (2 μs), repetition frequency (200 kHz), and duty cycle (40%). The fiber was guided using a stereotaxic instrument to precisely approach the target area of the auditory cortex (anteroposterior: −2.65 mm, mediolateral: −4.2 mm, dorsoventral: −2.65 mm).

The protocol for in vitro irradiation mirrors that of in vivo irradiation. The dish was positioned on the freely moving platform, controlled by software at a speed of 0.2 mm/s, with each scanning spot taking 2 s. Following irradiation, the cell culture dishes were incubated for 24 h for subsequent experiments.

### Statistical analysis

All data were expressed as mean ± standard error of the mean (SEM). The Student *t* test was used to compare normally distributed data. One-way and 2-way analyses of variance were used to compare data involving 3 or more groups. A 2-sided *P* < 0.05 was considered statistically significant.

## Data Availability

Data supporting the findings of this study are available from the corresponding author upon reasonable request.
