# Supplementary material for: Mid-Infrared Photons Alleviate Tinnitus by Activating the KCNQ2 Channel in the Auditory Cortex
Source: Research (Wash D C). 2024 Sep 18;7:0479. doi: 10.34133/research.0479 (PMC11408936; doi:10.34133/research.0479)
Supplement: Supplementary 1 — Figs. S1 to S9 [file research.0479.f1.zip › supplementary material.docx]

Supplementary Information for

**Mid-infrared photons alleviate tinnitus through activating KCNQ2 channel in the auditory cortex**

**Methods**

**Golgi staining**

Mice were euthanized with isoflurane and the brain was immediately removed. Brains were gently rinsed several times with normal saline and submerged in Golgi–Cox staining solution (Servicebio, Wuhan, China). The tissue was then placed in a cool and ventilated place in the dark for 14 days (2-day and 4 3-day soak). After immersion in distilled water three times, the tissue was treated overnight with 80% glacial acetic acid until it was soft and subsequently placed in 30% sucrose. The tissue was sectioned (100 µm) using a vibratome (LEICA, VT1000S), and slides were pasted, and dried on a gelatin slide in the dark overnight. After being soaked in ammonia water and an acid-hardening fixing solution, the tissue slides were dried and sealed with glycerin gelatin. Golgi-labeled neurons were visualized using a Nikon Eclipse E100 microscope and panoramic images of the brain tissue were obtained using Pannoramic 250 multi-layer scanning with a digital slice scanner.

For linear and semilog Sholl analysis, 10 µm spaced concentric circles centered at cell body were applied to images using the ImageJ software. Intersections between dendrite and circles were counted (linear Sholl analysis) and the log of number of intersections within each circle was calculated (semilog Sholl analysis)[1, 2]. Total dendrite length was measured using the Neuro J plugin. Spines (mushroom, thin, stubby, and filopodia) were identified using the criteria described earlier[3]. A randomly selected basal dendrite was used to measure the spine density[4].

**Transmission electron microscopy**

The brain tissue (<1mm^3^) within the mouse auditory cortex was harvested immediately after euthanasia with isoflurane. The tissue blocks were fixed with fresh transmission electron microscopy (TEM) fixative (Servicebio, Wuhan, China) and then incubated with 1% OsO_4_ in 0.1 M phosphate buffer (pH 7.4) for 2 h at room temperature (RT). After gradient dehydration with ethanol, blocks were embedded in resin. The resin blocks were cut to 60–80 nm thickness using an ultramicrotome and stained with a 2% uranium acetate saturated alcohol solution in the dark. The slides were then rinsed with ultrapure water and stained with 2.6% lead citrate. After drying with a filter paper, cuprum grids with micro brain slides were placed on a grid board and dried overnight at RT. A Hitachi 7800 electron microscope was used to obtain electron micrographs at ×11,000 or ×30,000 magnification.

Electron micrographs (×11,000) were randomly captured to assess the density of the excitatory synapses. A high postsynaptic density (PSD), wide synaptic cleft, and round presynaptic vesicles were the criterions for identifying excitatory synapses and postsynaptic sites.[5] The density was calculated as the number of synapses per unit area of each image.[6] Images (×30,000) were analyzed as previously described to determine the specific properties of each synapse (number of synaptic vesicles, PSD thickness, synaptic cleft width, synaptic interface curvature, and length of the synaptic active zone).[7]

**Patch clamp recording**

The brain slices were transferred to an incubation chamber, where they were immersed in a recording solution containing (in mM) 115 NaCl, 5 KCl, 1.25 Na2HPO4, 10 glucose, 2 pyruvate, 25 NaHCO3, 2 MgCl2, and 2 CaCl2, adjusted to pH 7.3–7.4 at room temperature (RT) and continuously oxygenated with 95% O2 and 5% CO2. The target area was identified using IR-DIC optics (10× magnification). Subsequently, the objective lens was switched to 40× magnification to locate active cells in the auditory cortex. Glass electrodes (5–7 MΩ) were filled with a solution containing (in mM) 130 potassium gluconate, 10 KCl, 10 HEPES, 1 MgCl2, 5 EGTA, 1 CaCl2, 2 Na2ATP, and 0.5 Na3GTP, adjusted to pH 7.4 (all chemicals from Fluka, NY, USA) were used for signal detection. Whole-cell recordings were conducted using Multiclamp 700 B amplifiers (Molecular Devices, USA) and a 1550s A/D board (Molecular Devices, USA).

For the recording of membrane potential responses to negative current injections, the step current by 20 pA from 0 to -100 pA over 500 ms was applied. The input resistance (Rin) was determined by the slope of the line fitted to voltage versus current relationship.

**Immunofluorescence**

For primary cell staining, the culture medium was aspirated from the cells and washed three times with PBS for 10 minutes each. Subsequently, a 20% formaldehyde sucrose solution was prepared and added to the ibidi petri dish for fixation. After washing three times with PBS-Triton X solution for 10 minutes each, the cells were incubated with PBS-Triton X for 15 minutes. Next, PBS-Triton X containing 3% goat serum was used to block the cells for 1 hour. The culture dish was then incubated overnight (at 4℃) with a diluted MAP2 primary antibody (CST; Number: 8707S) in PBS-Triton X containing 1% goat serum (1:200). Following this, the culture dish was washed three times with PBS-Triton X solution for 10 minutes each, and incubated at room temperature for 2 hours with Alexa Fluor 594 antibody (Invitrogen, A-11012, 1:500). After three washes with PBS-Triton X, the culture dish was covered with an antifade solution containing DAPI. Fluorescent images (20x) were captured using a Leica microscope.

For tissue staining, the mice were anesthetized with 1.5% isoflurane, perfused with cold saline, and fixed with 4% paraformaldehyde. The brain containing the auditory cortex was isolated and postfixed in the same fixative for 12 hours, followed by immersion in 30% sucrose for 48 hours. Tissue slices (30 mm) were obtained using a cryotome and pre-blocked with 5% donkey serum and 0.3% Triton X-100 for 2 hours at room temperature. The sections were then incubated with the following primary antibodies at 4℃ for 12 hours: rabbit anti-KCNQ2 (1:500, GeneTex, GTX82891) and rabbit anti-KCNQ3 (1:500, GeneTex, GTX16228). Subsequently, the tissue slices were incubated with secondary donkey Alexa Fluor 488 (1:500, Invitrogen, R37118) for 2 hours at room temperature. The slices were photographed and viewed using confocal microscopy (LEICA, DMIL) with fluorescence to identify the neurons.

**q-PCR**

The experimental procedures for RNA extraction in this study followed the methods used in previous studies[8]. Total RNA (n = 3) was extracted using RNA Extraction Reagent (Servicebio, Wuhan, China) and converted into complementary DNA (cDNA) using the Servicebio RT First Strand cDNA Synthesis Kit. For qPCR analysis, 2× SYBR Green qPCR Master Mix (Servicebio) was used. The mRNA expression levels of the target genes were normalized to the expression levels of glyceraldehyde-3-phosphate dehydrogenase (GAPDH) mRNA, and the fold changes in expression differences were calculated using the 2−ΔΔCT method[9]. The sequences of the oligonucleotide primers (Servicebio) used in this study are provided in Table 1.

**RNA-seq**

The procedure for extracting total RNA from the auditory cortex of brain tissue remains the same as described above. The concentration and purity of the extracted samples are assessed using the NanoDrop 2000 and Agilent Bioanalyzer 2100 systems. Library preparation, clustering, and sequencing are performed on the Illumina NovaSeq 6000 platform. To ensure high-quality data, sequences containing joints, ploy-N sequences, and sequences of low quality are removed from the original data. The quantification of gene expression levels is determined by calculating the number of mapped fragments per kilobase of transcript per million. Differential expression analysis between the two groups is conducted using DESeq2. The corrected P-value is obtained using Benjamini and Hochberg's method to control the false discovery rate. Genes analyzed by DESeq2 with a P-value less than 0.01 and a fold change of at least two after correction are identified as differentially expressed.

**Western Blot**

After rapidly decapitating the animals, the tissue from the right auditory cortex region was promptly collected. The tissue surface was washed with PBS buffer to eliminate any remaining blood, and then the tissue was swiftly placed in a centrifuge tube. A pre-prepared lysate solution, consisting of RIPA lysate, phosphatase inhibitor, protease inhibitor, and PMSF, was added to the tube. As for the cells, after washing them with PBS buffer, ice-cold RIPA buffer was added to the dish. The adherent cells were then scraped off the dish and transferred into a microcentrifuge tube. The total proteins, derived from both the tissue and the cells, were transferred onto a polyvinylidene fluoride membrane. The proteins were subsequently blocked with 5% skim milk for 2 hours at room temperature, and then incubated overnight at 4 ℃ with primary antibodies, including KCNQ2 (GeneTex, GTX82891), KCNQ3 (GeneTex, GTX16228), GAPDH (Servicebio, GB15002), and Actin (Servicebio, GB11001). To obtain the corresponding KCNQ2 and KCNQ3 protein bands, appropriate secondary antibodies (horseradish peroxide-coupled goat anti-rabbit, GB23303; horseradish peroxide-coupled goat anti-mouse, GB23301) were used. GAPDH and Actin were employed as internal controls.

**Primary cell culture**

The primary neuronal culture was conducted following a previous study with some modifications[10]. To begin, the cerebral cortex was carefully isolated from C57 mice at postnatal day 0 and rinsed with PBS. The tissue was then dissected using ophthalmic scissors and digested with a solution consisting of 0.3ml Trypsin/EDTA, 0.3ml PBS, and 2μl DNase per animal. This digestion process took place at 37℃ for 30 minutes, with shaking every 5 minutes to ensure complete digestion. Subsequently, the tissues were centrifuged at 1000 rpm for 5 minutes, the supernatant was discarded, and the cells were resuspended. For further seeding, a low cell density of 20000 cells/cm2 was used in an ibidi culture dish that had been pre-coated with Poly-D-Lysine. A medium containing NB (Gibco, 21103049), B27 (Gibco, 17504001), glutamine (Gibco, 17504001), and penicillin-streptomycin (Gibco, 17504001) was used to support the growth of the cultured neurons. Regular observations were made to monitor the growth of the neurons, and the medium was changed every 2 days, with half of the liquid being replaced each time.

**Reference**

1. Sholl DA. Dendritic organization in the neurons of the visual and motor cortices of the cat. Journal of anatomy. 1953; 87: 387-406.

2. Jacobs S, Cheng C, Doering LC. Hippocampal neuronal subtypes develop abnormal dendritic arbors in the presence of Fragile X astrocytes. Neuroscience. 2016; 324: 202-17.

3. Medalla M, Luebke JI. Diversity of glutamatergic synaptic strength in lateral prefrontal versus primary visual cortices in the rhesus monkey. The Journal of neuroscience : the official journal of the Society for Neuroscience. 2015; 35: 112-27.

4. González-Burgos G, Miyamae T, Krimer Y, Gulchina Y, Pafundo DE, Krimer O, et al. Distinct Properties of Layer 3 Pyramidal Neurons from Prefrontal and Parietal Areas of the Monkey Neocortex. The Journal of neuroscience : the official journal of the Society for Neuroscience. 2019; 39: 7277-90.

5. Medalla M, Gilman JP, Wang JY, Luebke JI. Strength and Diversity of Inhibitory Signaling Differentiates Primate Anterior Cingulate from Lateral Prefrontal Cortex. The Journal of neuroscience : the official journal of the Society for Neuroscience. 2017; 37: 4717-34.

6. Mohan V, Wade SD, Sullivan CS, Kasten MR, Sweetman C, Stewart R, et al. Close Homolog of L1 Regulates Dendritic Spine Density in the Mouse Cerebral Cortex Through Semaphorin 3B. The Journal of neuroscience : the official journal of the Society for Neuroscience. 2019; 39: 6233-50.

7. Xiao Y, Fu H, Han X, Hu X, Gu H, Chen Y, et al. Role of synaptic structural plasticity in impairments of spatial learning and memory induced by developmental lead exposure in Wistar rats. PloS one. 2014; 9: e115556.

8. Fang J, Wang H, Zhou J, Dai W, Zhu Y, Zhou Y, et al. Baicalin provides neuroprotection in traumatic brain injury mice model through Akt/Nrf2 pathway. Drug design, development and therapy. 2018; 12: 2497-508.

9. Livak KJ, Schmittgen TD. Analysis of relative gene expression data using real-time quantitative PCR and the 2(-Delta Delta C(T)) Method. Methods (San Diego, Calif). 2001; 25: 402-8.

10. Sethi S, Keil KP, Chen H, Hayakawa K, Li X, Lin Y, et al. Detection of 3,3'-Dichlorobiphenyl in Human Maternal Plasma and Its Effects on Axonal and Dendritic Growth in Primary Rat Neurons. Toxicological sciences : an official journal of the Society of Toxicology. 2017; 158: 401-11.


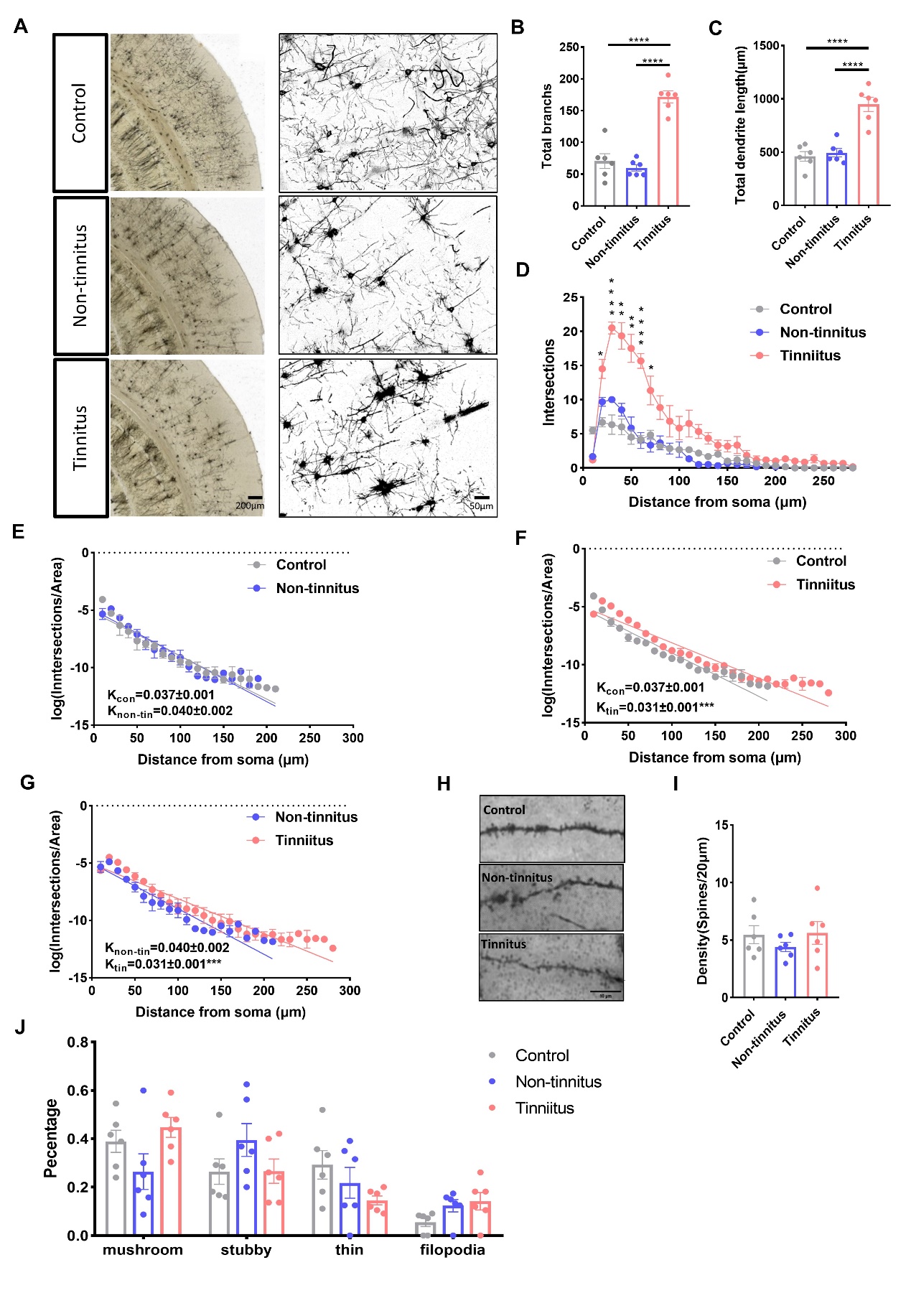


Figure S1: Tinnitus increases the complexity of pyramidal neurons in the auditory cortex, but not their spines.

(A) Representative Golgi staining images of pyramidal neurons in the control, non-tinnitus, and tinnitus groups. (B, C) The tinnitus group showed a higher number of branches and longer dendrites compared to the non-tinnitus and control groups. (D) Linear analysis of dendritic branches in the tinnitus, non-tinnitus, and control groups.

(E-G) Semi-logarithmic analysis of dendritic branches in the tinnitus, non-tinnitus, and control groups. (H) Representative images of dendritic spines in the non-tinnitus, tinnitus, and control groups. (I) Spine density of neurons in the control, non-tinnitus, and tinnitus groups. (J) Distribution of spine subtypes in the tinnitus, non-tinnitus, and control groups. N=3 animals; **p*<0.05, ***p*<0.01, ****p*<0.001; error bars represent standard error of the mean.


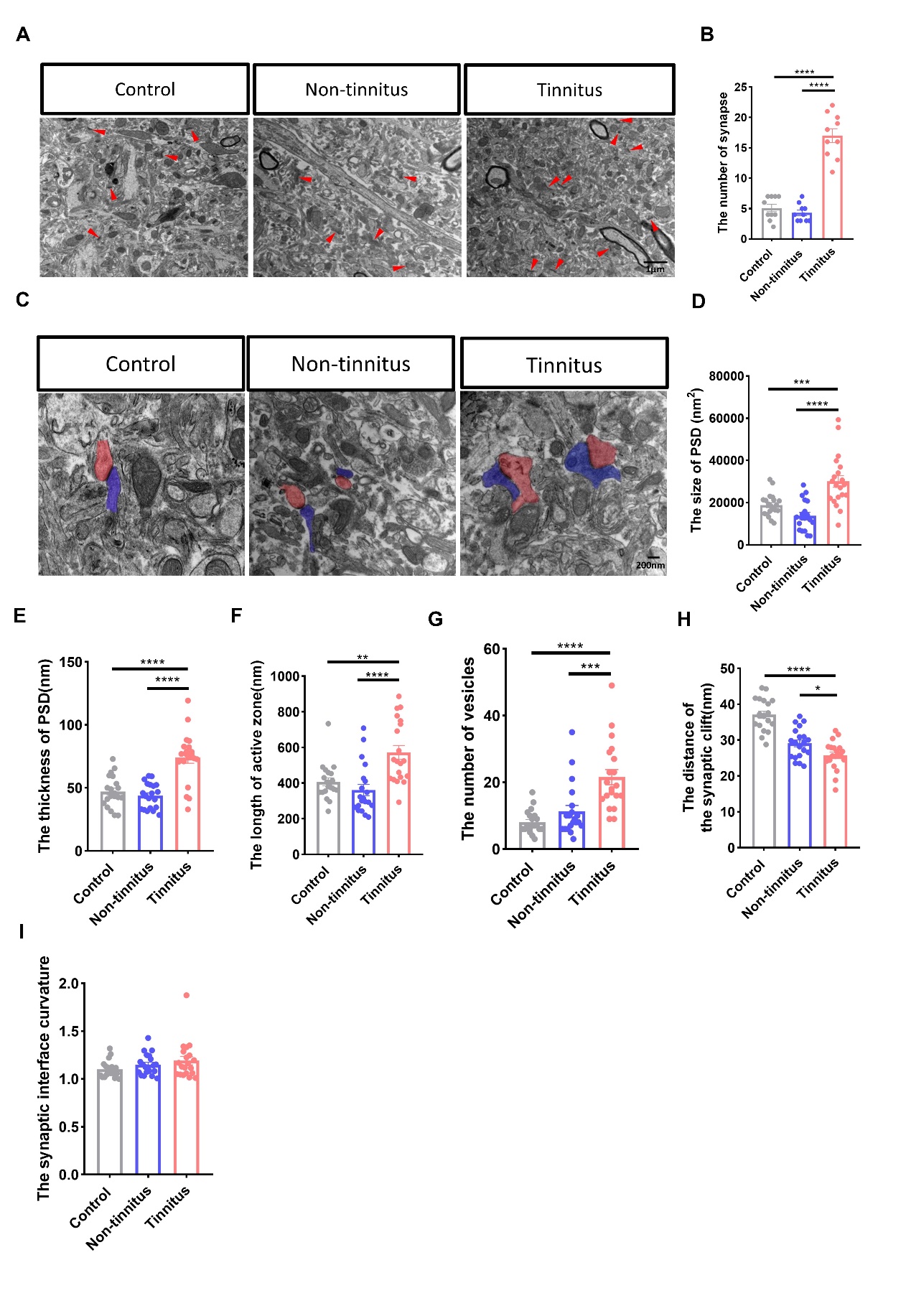


Figure S2: Tinnitus enhances information transmission between neighboring neurons.

(A) Representative images showing synapses in the control, non-tinnitus, and tinnitus groups. (B) Bar chart illustrating the number of synapses in the control, non-tinnitus, and tinnitus groups. (C) Representative images displaying the structural characteristics of synapses in the control, non-tinnitus, and tinnitus groups. Comparison of synaptic parameters between the control, non-tinnitus, and tinnitus groups, including the size of the postsynaptic density (PSD) (D), thickness of the PSD (E), length of the active zone (F), number of vesicles (G), distance of the synaptic cleft (H), and curvature of the synaptic interface (I). N=3 animals; **p*<0.05, ***p*<0.01, ****p*<0.001, *****p*<0.0001; error bars represent standard error of the mean.


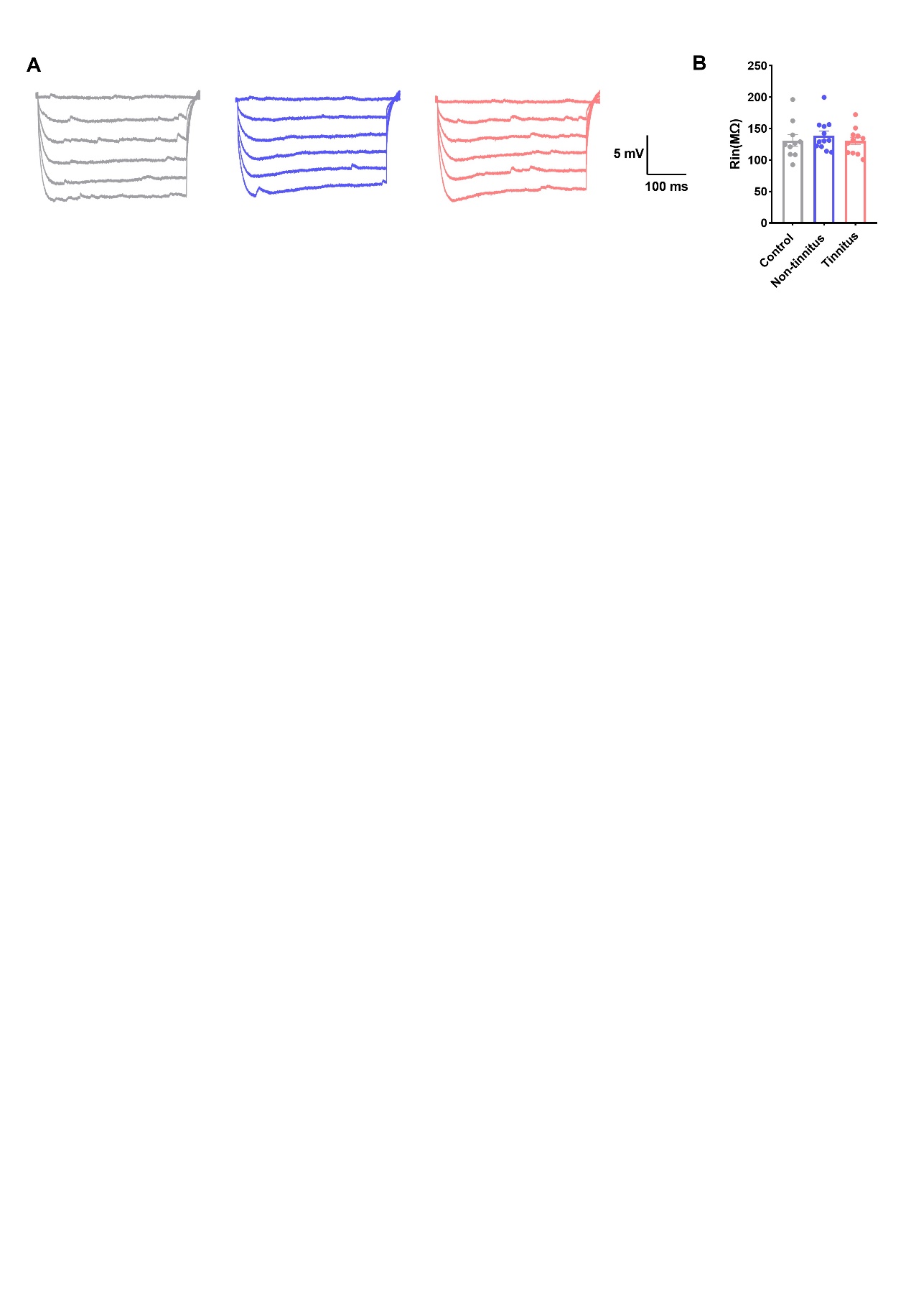


Figure S3: Influence of tinnitus on the membrane potential responses to negative current injections.

(A) Voltage responses to various current injection steps in pyramidal neurons from either control, non-tinnitus and tinnitus mice. (B) Summary graphs showing the effect of tinnitus on the input resistance. N=10-12 cells; error bars represent standard error of the mean.


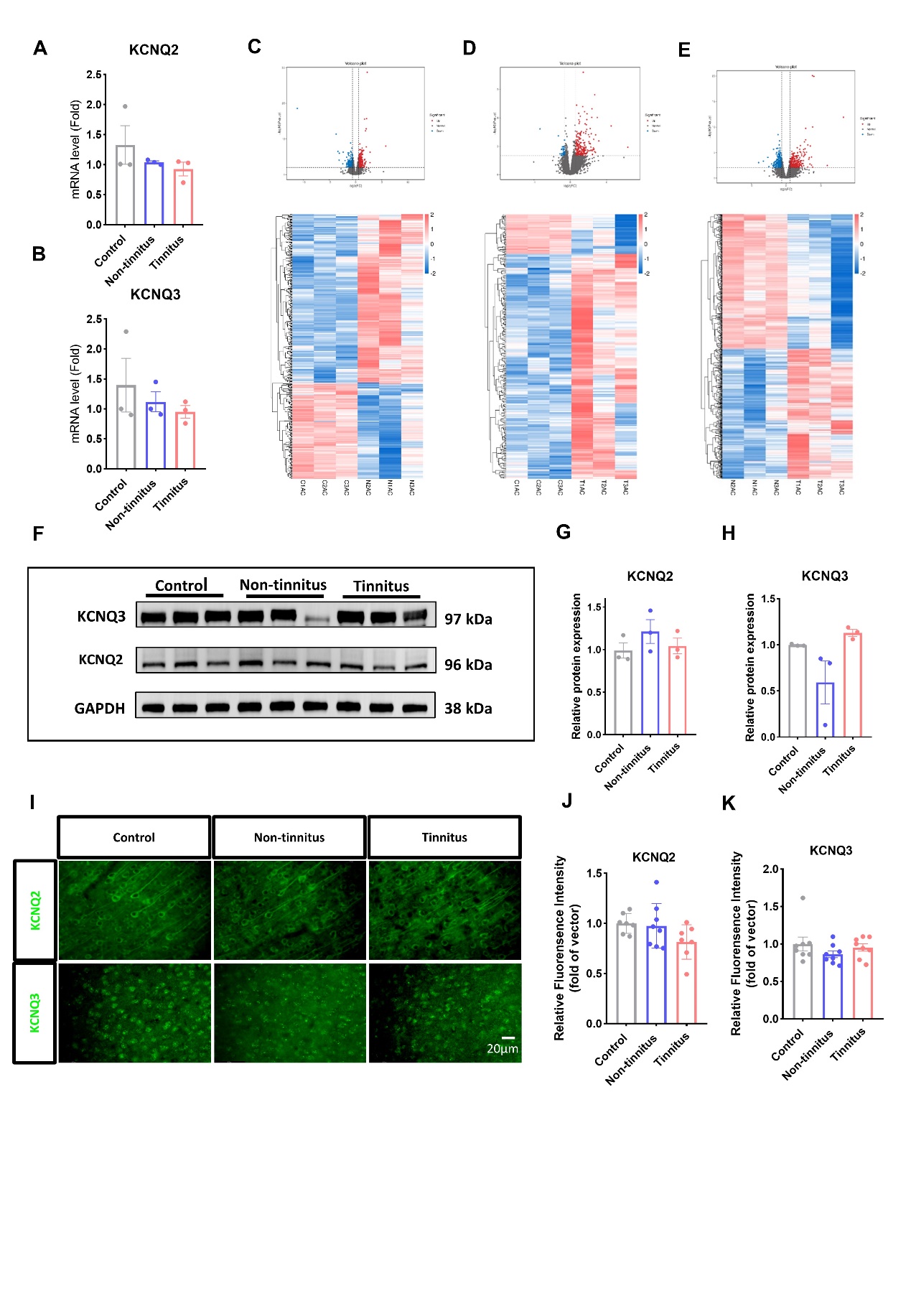


Figure S4: Changes in transcription and translation of KCNQ2 and KCNQ3 following tinnitus.

(A) mRNA levels of KCNQ2 in the control, non-tinnitus, and tinnitus groups. (B) mRNA levels of KCNQ3 in the control, non-tinnitus, and tinnitus groups. (C) Differentially expressed genes between the control and non-tinnitus groups. (D) Differentially expressed genes between the control and tinnitus groups. (E) Differentially expressed genes between the non-tinnitus and tinnitus groups. (F) Expression of KCNQ2 and KCNQ3 proteins in the control, non-tinnitus, and tinnitus groups. (G) Bar chart showing the relative protein expression of KCNQ2. (H) Bar chart showing the relative protein expression of KCNQ3. (I) Fluorescent image of KCNQ2 and KCNQ3 proteins in the control, non-tinnitus, and tinnitus groups. (J) Bar chart showing the relative fluorescence intensity of KCNQ2. (K) Bar chart showing the relative fluorescence intensity of KCNQ3. N=3 animals; **p*<0.05, ***p*<0.01, ****p*<0.001, *****p*<0.0001; error bars indicate the standard error of the mean.


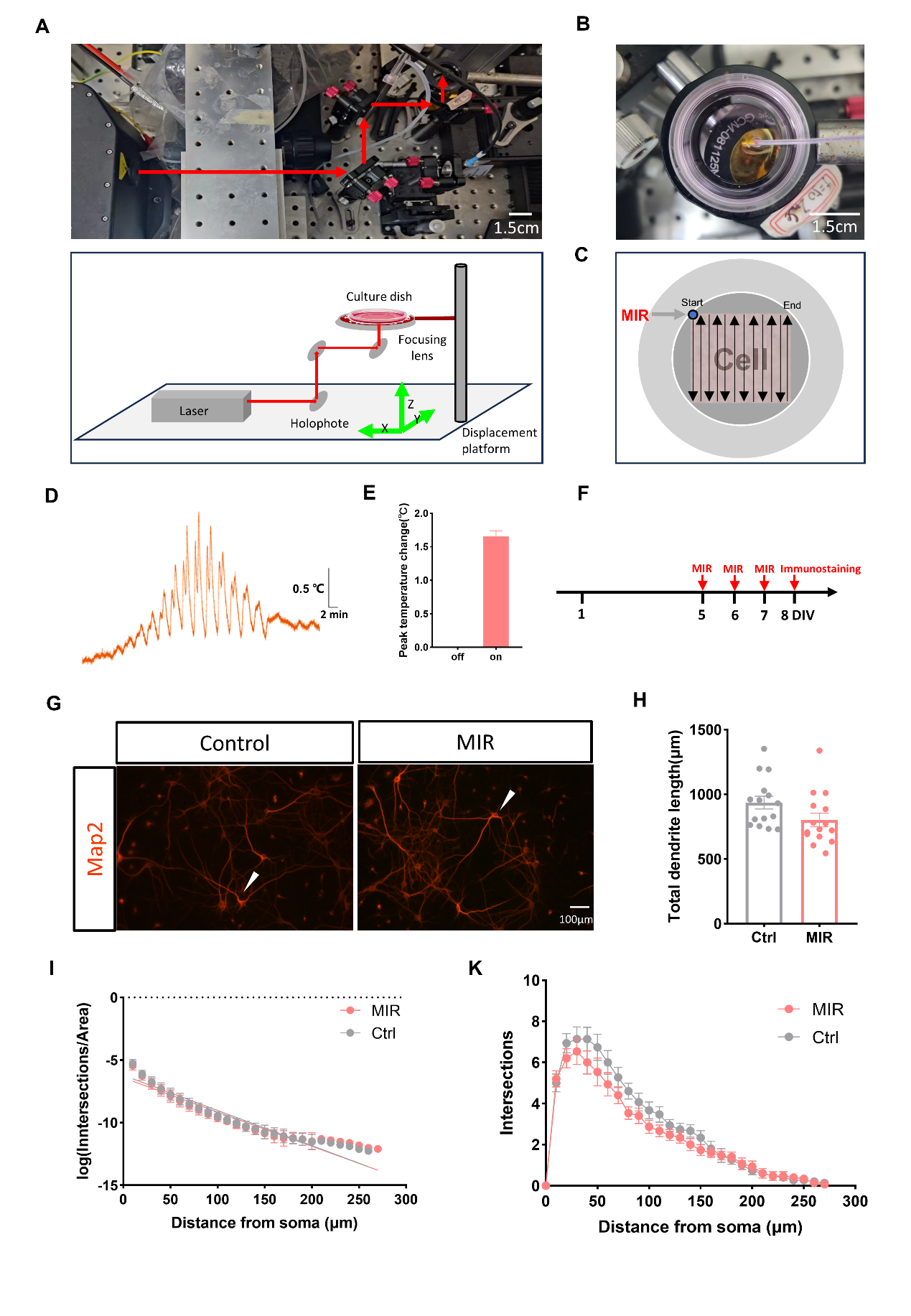


Figure S5: Effect of MIR on neuronal arborization.

(A) Image of MIR irradiation for cultured cells. (B) Image of the position between the temperature-sensitive probe and the culture dish. (C) Schematic image of cell irradiation. (D) Concrete temperature changes during MIR irradiation. (E) Bar chart showing the temperature changes during MIR irradiation. (F) Experimental design of cell irradiation. (G) Arborization of cultured cortical neurons in the control and MIR groups. (H) Bar chart showing the total dendrite length between the control and MIR groups. (I) Semi-log Sholl analysis of dendrites between the control and MIR groups. (J) Linear Sholl analysis of dendrites between the control and MIR groups. N=3 dishes; **p*<0.05, ***p*<0.01, ****p*<0.001, *****p*<0.0001; error bars indicate the standard error of the mean.


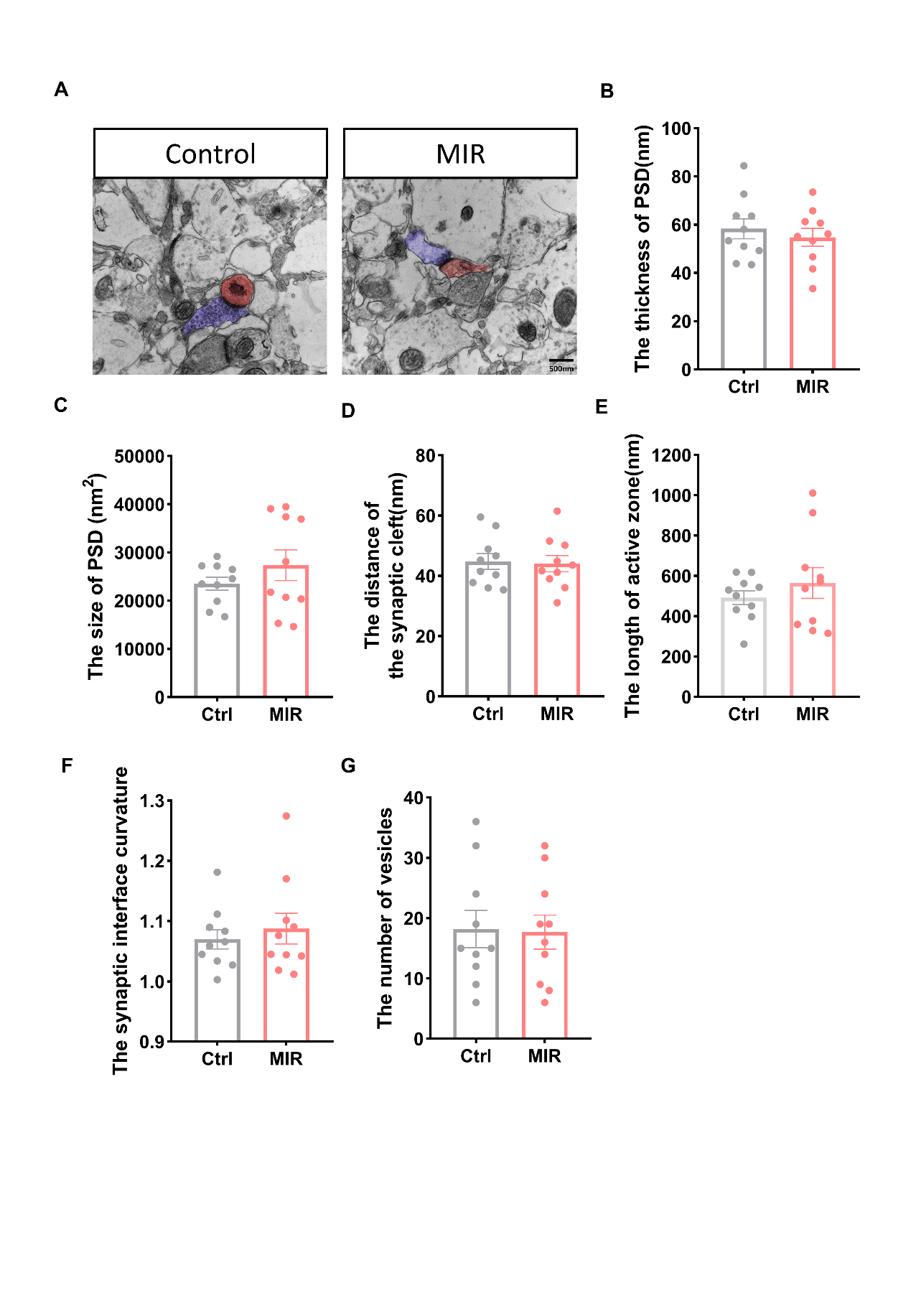


Figure S6: Effect of MIR on the structure of synapse.

(A) Image of synaptic structure in the control and MIR group. (B) Bar chart of the thickness of PSD between control and MIR group. (C) Bar chart of the size of PSD between control and MIR group. (D) Bar chart of the distance of the synaptic cleft between control and MIR group. (E) Bar chart of the total length of active zone between control and MIR group. (F) Bar chart of the synaptic interface curvatures between control and MIR group. (G) Bar chart of the number of vesicles between control and MIR group. N=3; **p*<0.05, ***p*<0.01, ****p*<0.001, *****p*<0.0001; error bars indicate standard error of mean.


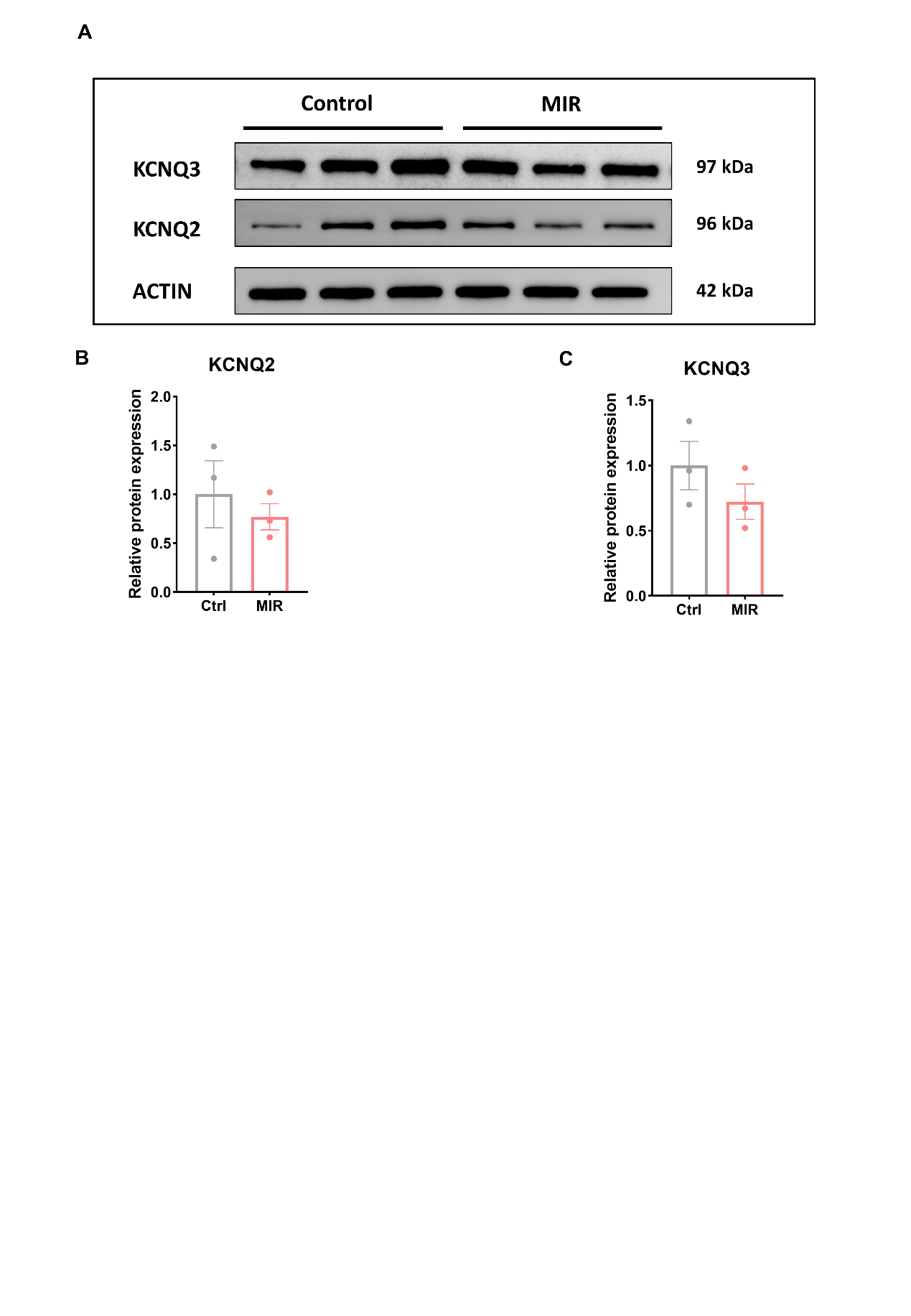


Figure S7: Effects of MIR on the expression of KCNQ2 and KCNQ3 proteins.

(A) Protein expression of KCNQ2 and KCNQ3 in the control and MIR group. (B) Bar chart showing the relative protein expression of KCNQ2. (C) Bar chart showing the relative protein expression of KCNQ3. N=3 dishes; **p*<0.05, ***p*<0.01, ****p*<0.001, *****p*<0.0001; error bars represent the standard error of the mean.


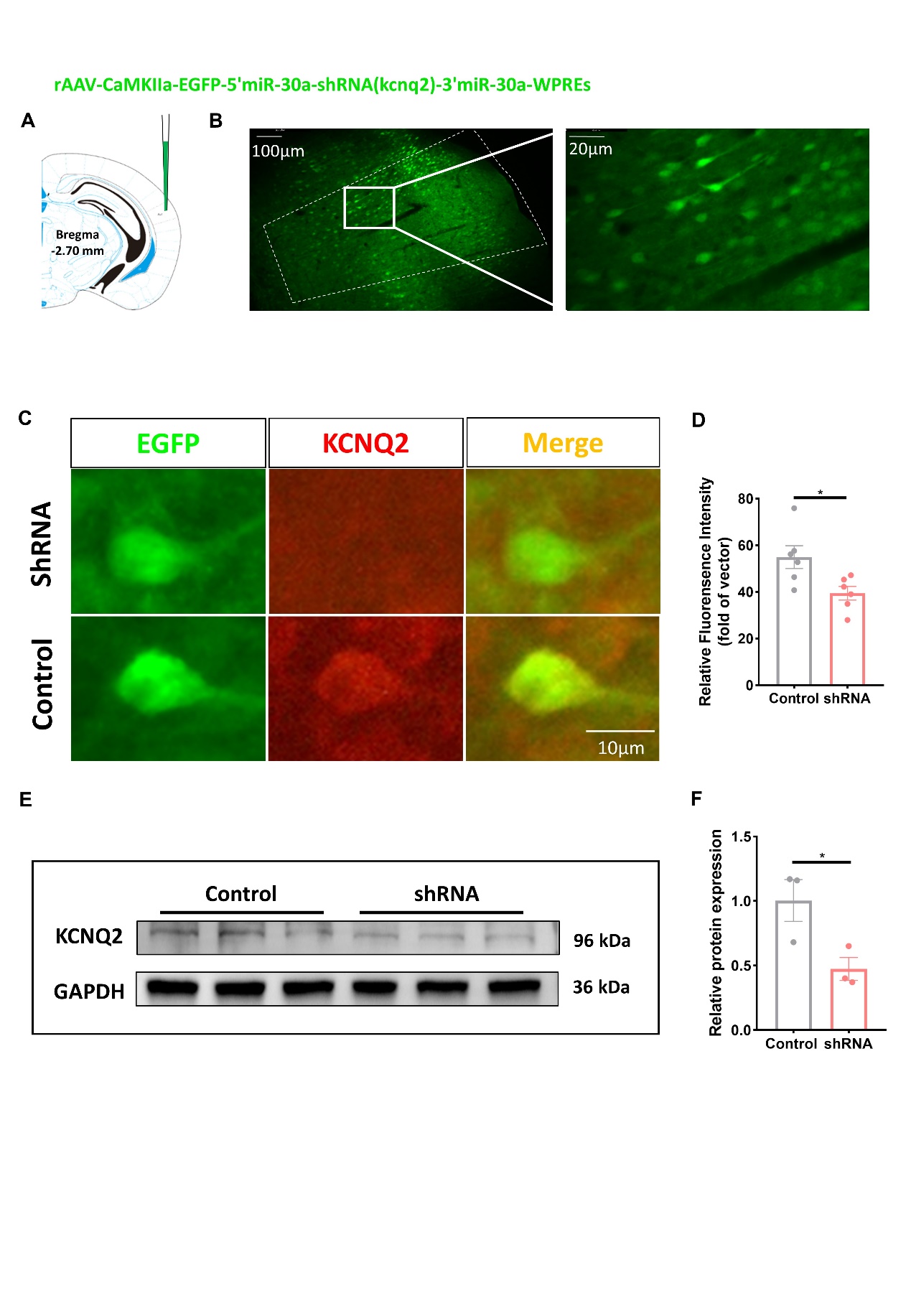


Figure S8: Effects of KCNQ2 virus on protein expression.

(A) Schematic representation of the target area in the auditory cortex. (B) Image showing neurons in the auditory cortex successfully infected with KCNQ2 virus. (C) Fluorescent image of neurons in the shRNA group and control group. (D) Bar chart showing the relative fluorescence intensity of KCNQ2 protein. (E) Image showing the specific protein expression of KCNQ2 in the control and shRNA group. (F) Bar chart showing the relative protein expression of KCNQ2 in the control and shRNA group. N=3 animals; **p*<0.05, ***p*<0.01, ****p*<0.001, *****p*<0.0001; error bars represent the standard error of the mean.


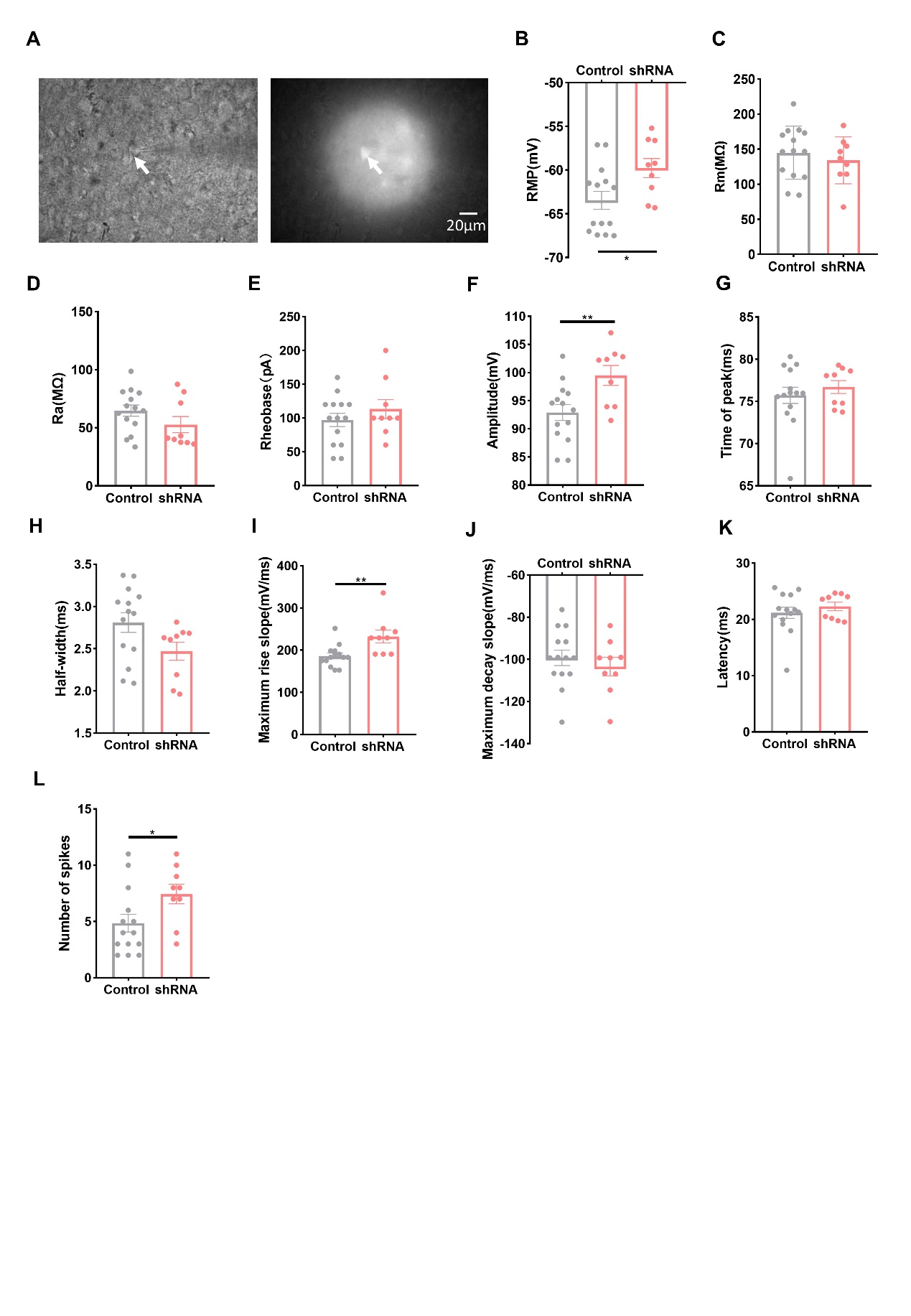


Figure S9: Effects of KCNQ2 virus on neural excitability.

(A) Schematic image of recorded neurons. (B-F) Comparison of negative properties, including resting membrane potential (RMPs) (B), membrane resistance (Rm) (C), axial resistance (Ra) (D), and rheobase (E), between the control and shRNA group. (F-L) Changes in positive properties between the control and shRNA group, including amplitude (F), time of peak (G), half-width (H), maximum rise slope (I), maximum decay slope (J), latency (K), and firing number (L) of action potentials. N=3 animals; **p*<0.05, ***p*<0.01, ****p*<0.001, *****p*<0.0001; error bars represent the standard error of the mean.
