# Supplementary figures and images for: Mid-Infrared Photons Alleviate Tinnitus by Activating the KCNQ2 Channel in the Auditory Cortex
Source: Research (Wash D C). 2024 Sep 18;7:0479. doi: 10.34133/research.0479 (PMC11408936; doi:10.34133/research.0479)

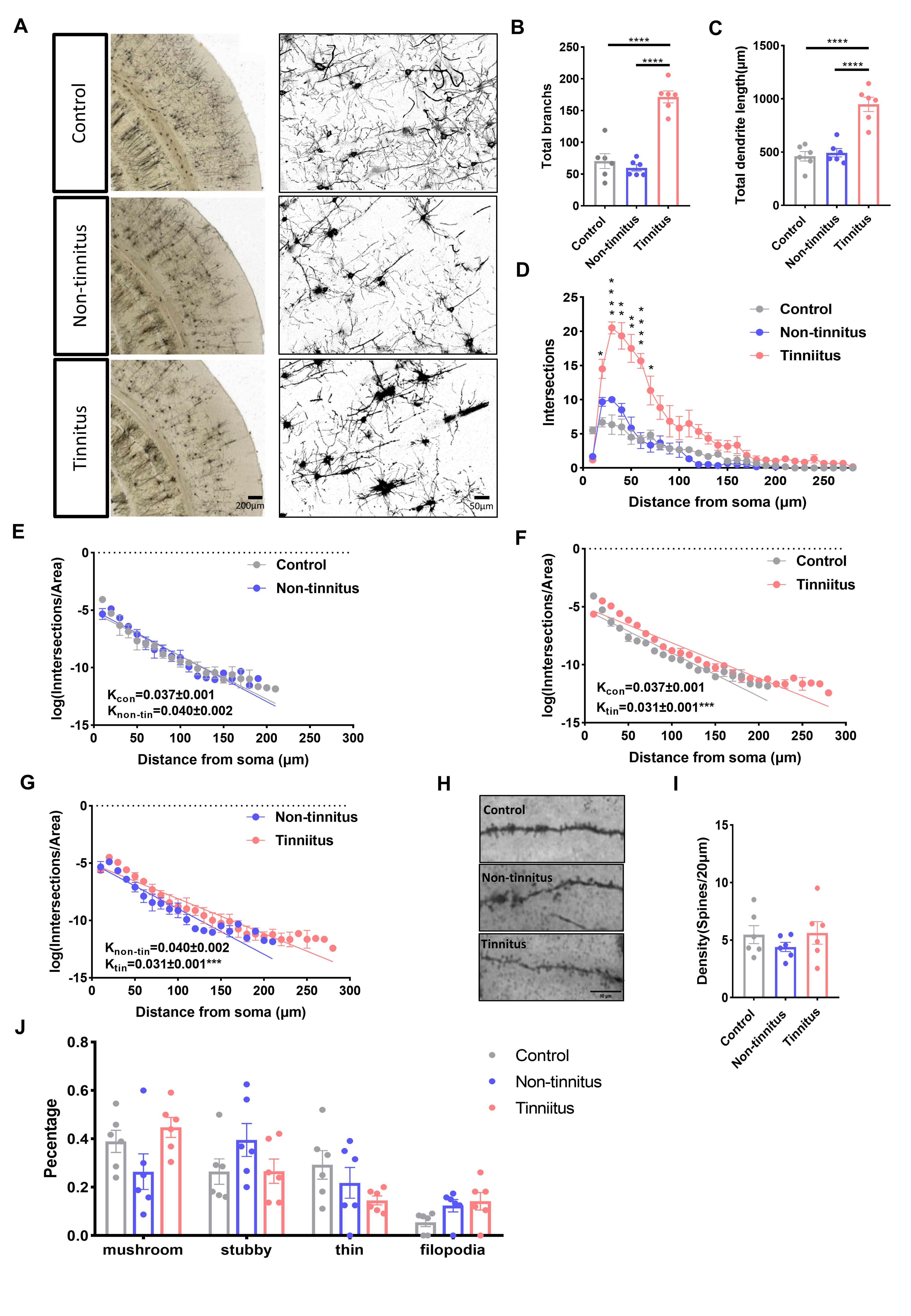

Supplement: Supplementary 1 — Figs. S1 to S9 [file research.0479.f1.zip › Figure S1.jpg]

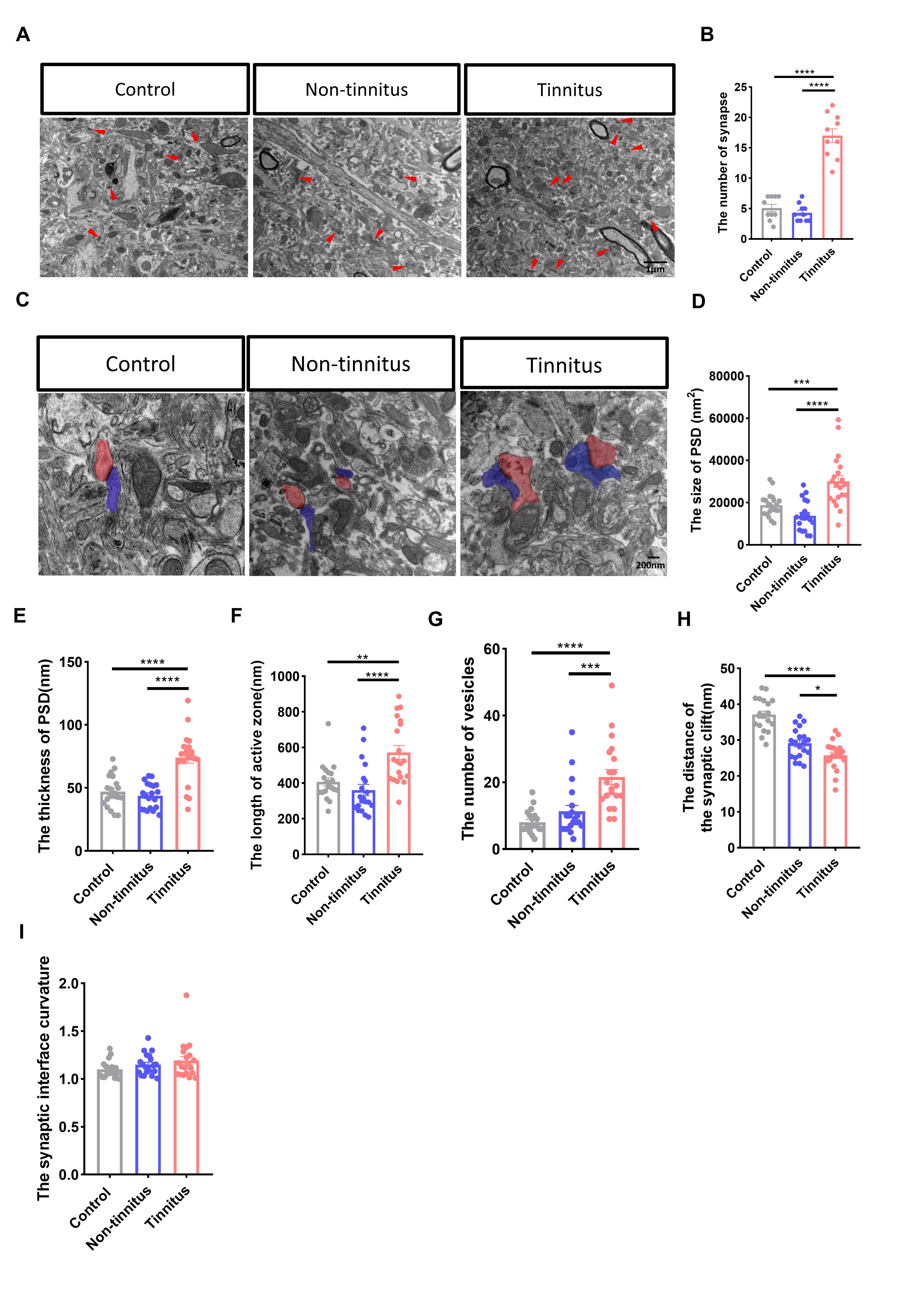

Supplement: Supplementary 1 — Figs. S1 to S9 [file research.0479.f1.zip › Figure S2.jpg]

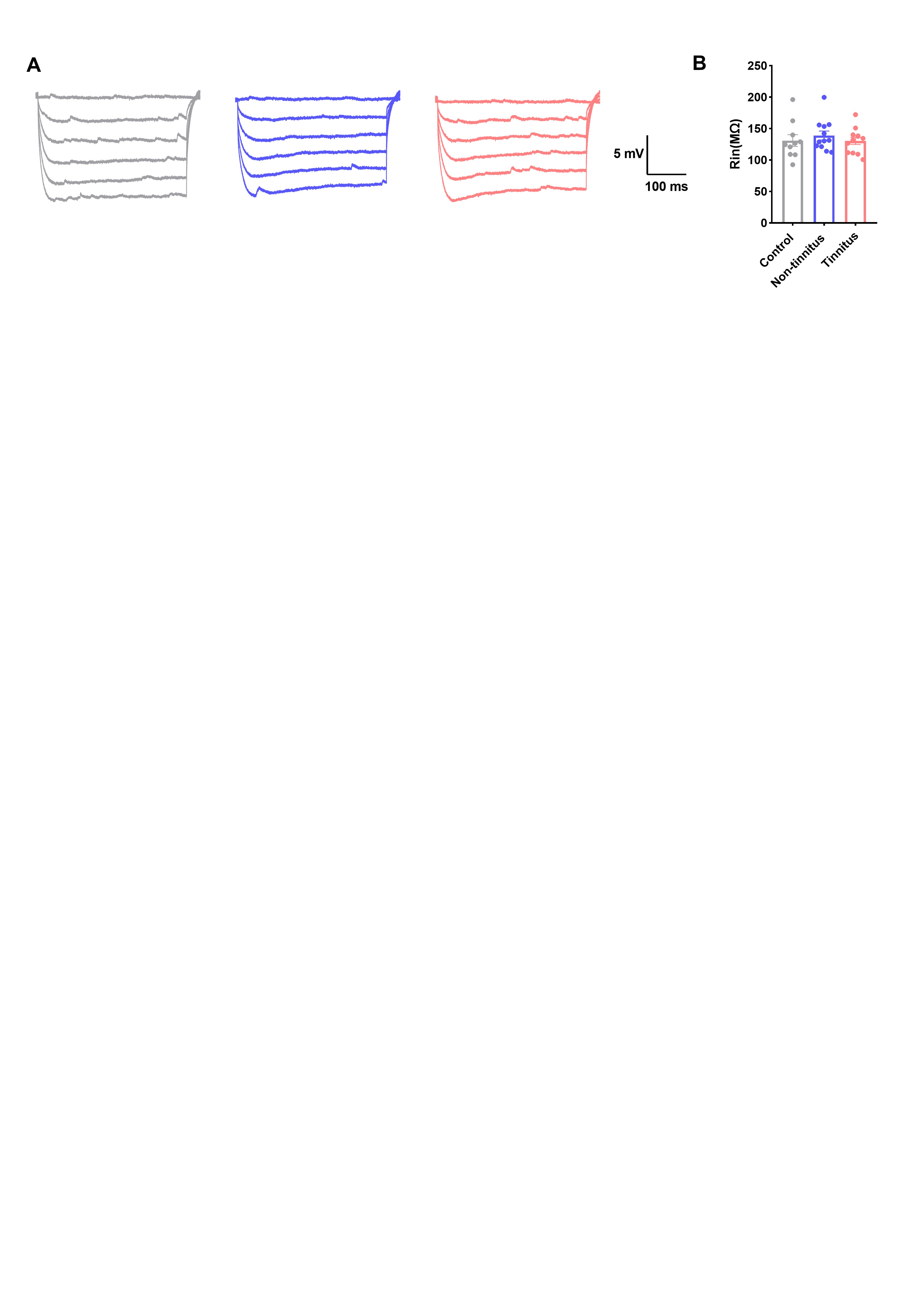

Supplement: Supplementary 1 — Figs. S1 to S9 [file research.0479.f1.zip › Figure S3.jpg]

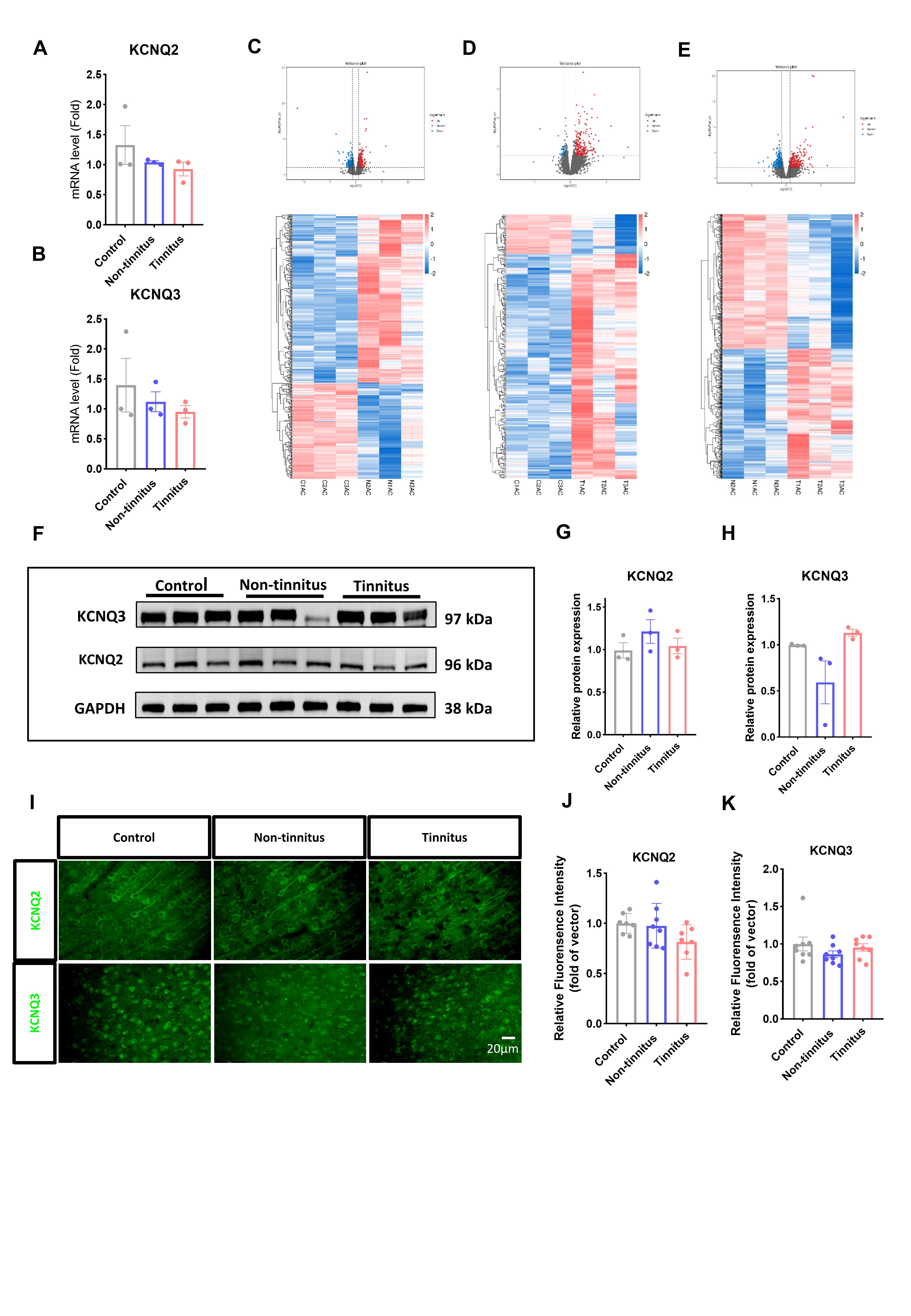

Supplement: Supplementary 1 — Figs. S1 to S9 [file research.0479.f1.zip › Figure S4.jpg]

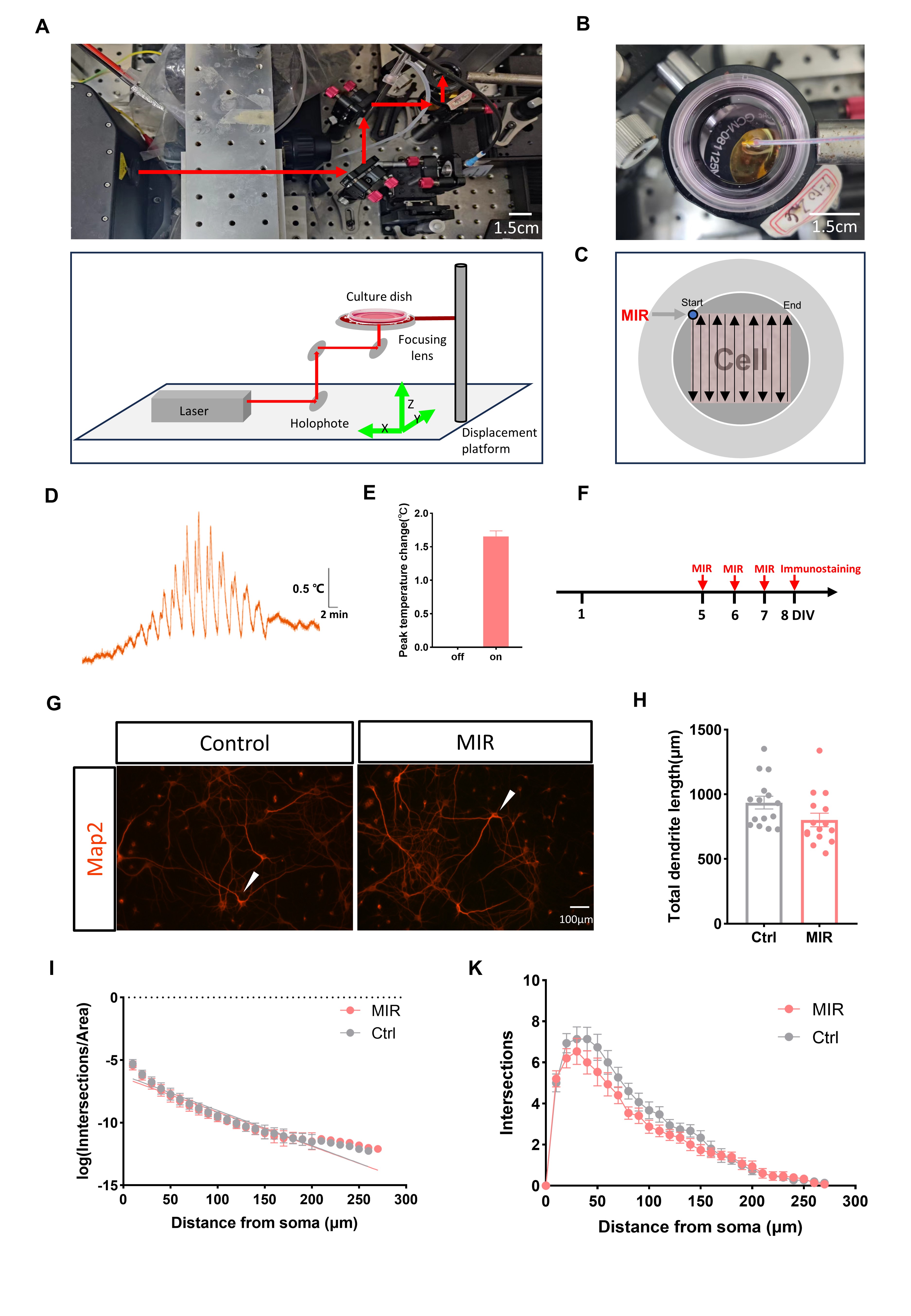

Supplement: Supplementary 1 — Figs. S1 to S9 [file research.0479.f1.zip › Figure S5.jpg]

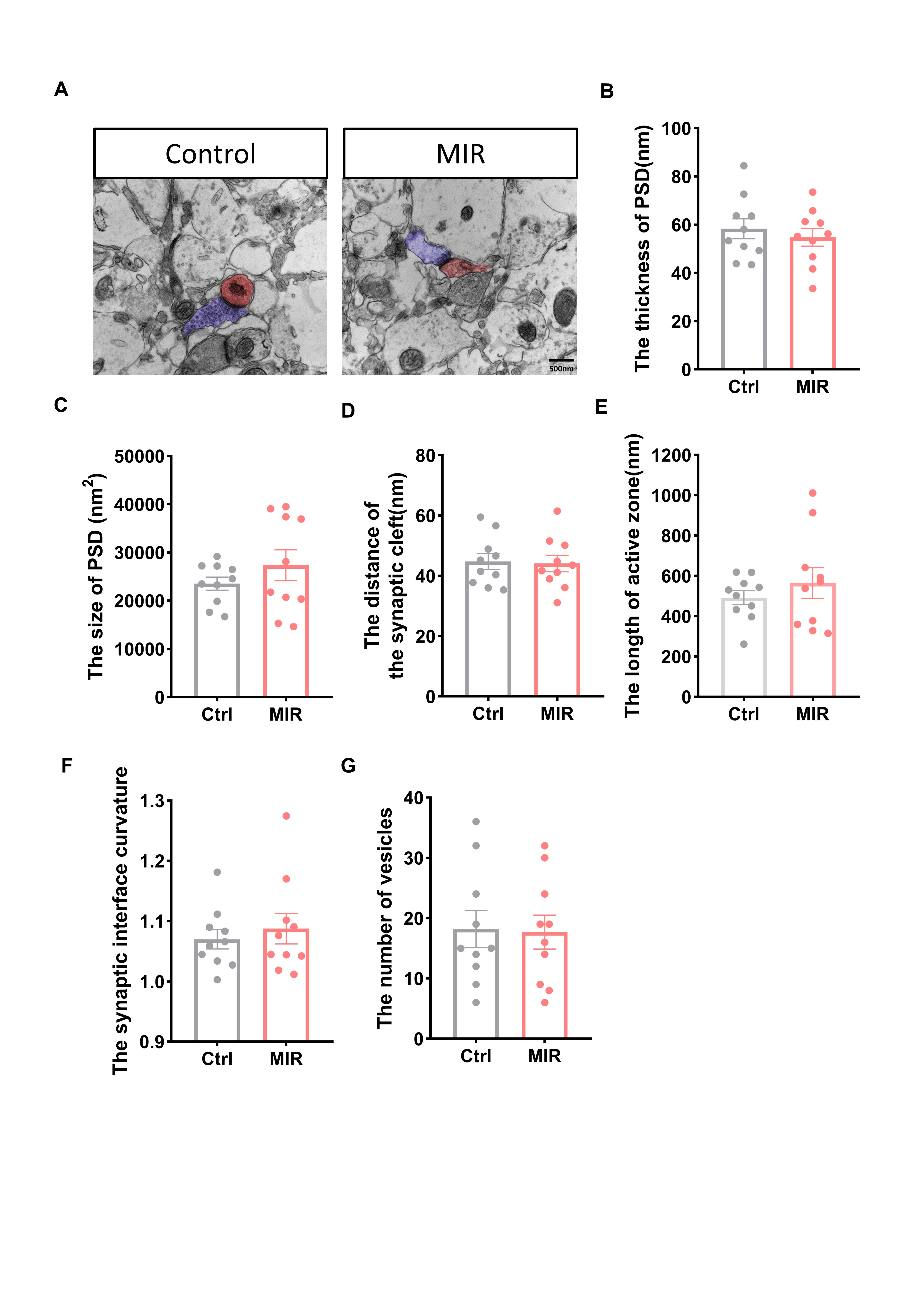

Supplement: Supplementary 1 — Figs. S1 to S9 [file research.0479.f1.zip › Figure S6.jpg]

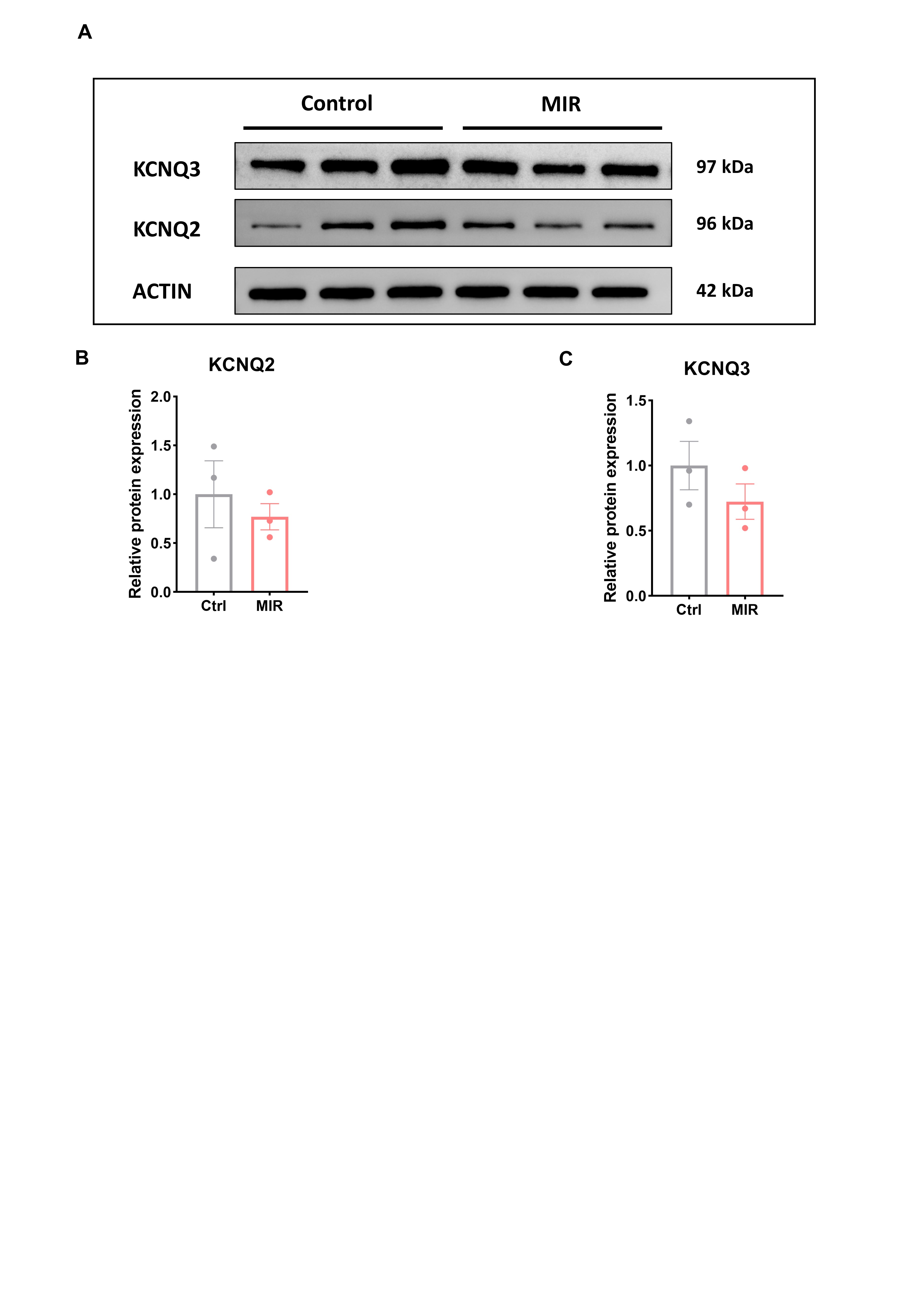

Supplement: Supplementary 1 — Figs. S1 to S9 [file research.0479.f1.zip › Figure S7.jpg]

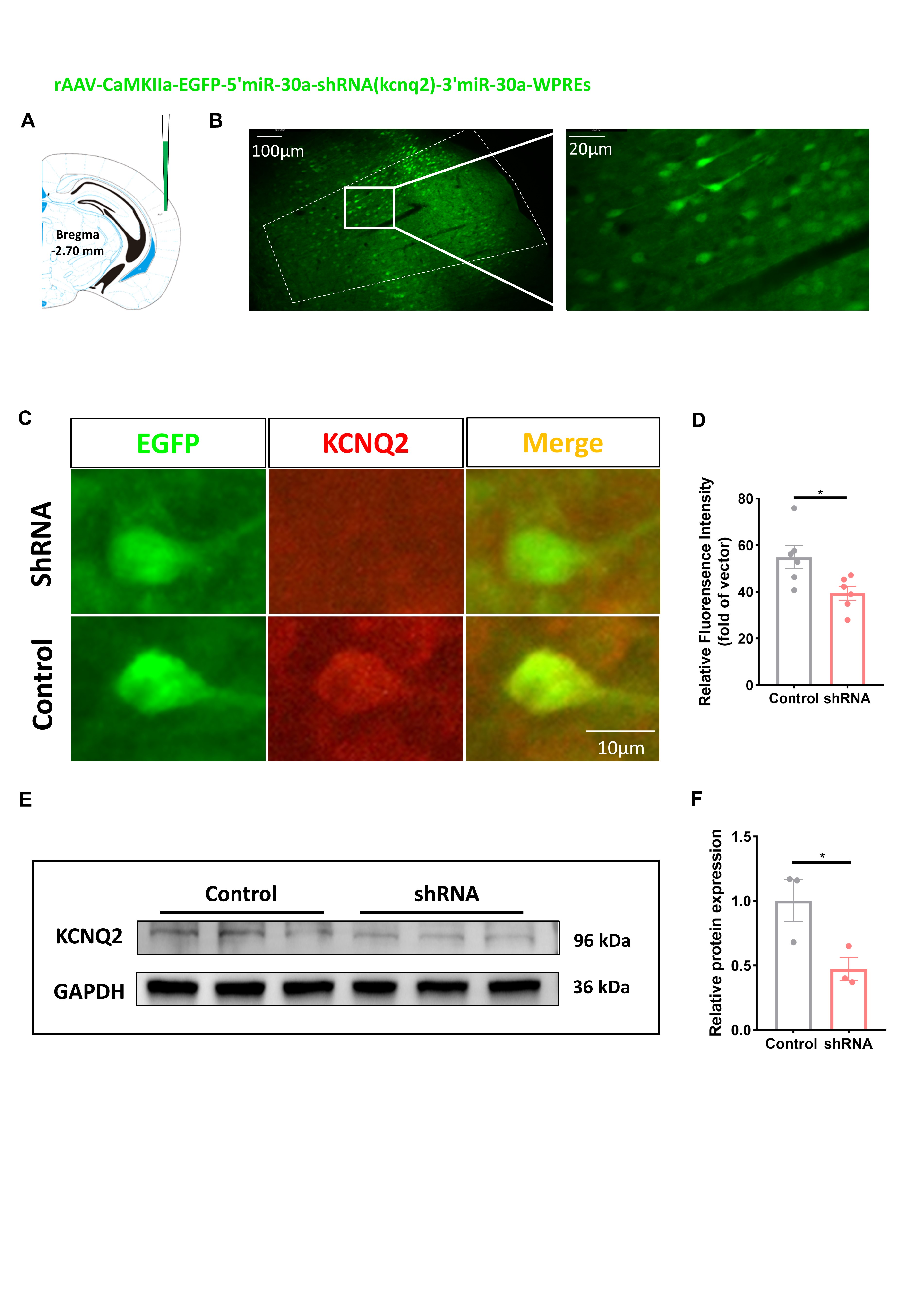

Supplement: Supplementary 1 — Figs. S1 to S9 [file research.0479.f1.zip › Figure S8.jpg]

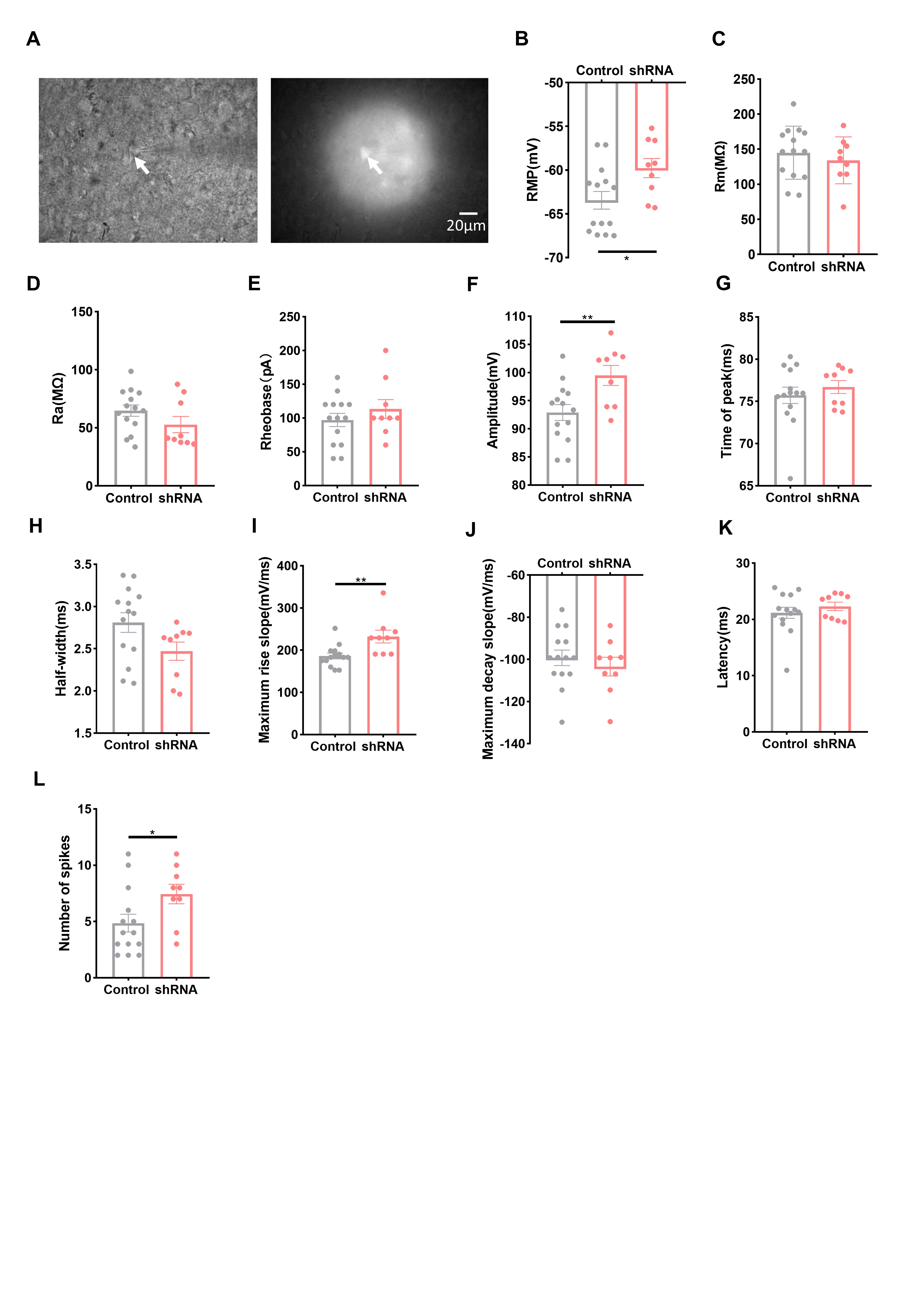

Supplement: Supplementary 1 — Figs. S1 to S9 [file research.0479.f1.zip › Figure S9.jpg]
